# Spatial navigation entropy suggests allocentric dysfunction in PPPD

**DOI:** 10.3389/fneur.2025.1599307

**Published:** 2025-05-16

**Authors:** Felipe Faúndez, Camilo Arévalo-Romero, Karen Villarroel, Claudio Lavín, Kevin Alarcón, Gustavo Vial, Francisco Artus, Pablo Billeke, Paul H. Delano, Hayo A. Breinbauer

**Affiliations:** ^1^Laboratory for Clinical Neuro-otology and Balance-Neuroscience, Department of Neuroscience, Facultad de Medicina, Universidad de Chile, Santiago, Chile; ^2^Departamento de Psicología, Universidad Autónoma de Chile, Santiago, Chile; ^3^Servicio de Otorrinolaringología Hospital Clínico San Borja Arriarán, Santiago, Chile; ^4^Laboratorio de Neurociencia Social y Neuromodulación, Centro de Investigación en Complejidad Social (neuroCICS), Facultad de Gobierno, Universidad del Desarrollo, Santiago, Chile; ^5^Centro Avanzado de Ingeniería Eléctrica y Electrónica, AC3E, Universidad Técnica Federico Santa María, Valparaíso, Chile; ^6^Servicio de Otorrinolaringología, Hospital Clínico Universidad de Chile, Santiago, Chile; ^7^Department of Otolaryngology, Facultad de Medicina Clínica Alemana, Universidad del Desarrollo, Santiago, Chile

**Keywords:** persistent postural perceptual dizziness, functional dizziness, spatial navigation, spatial cognition, functional neurological disorder, chronic dizziness

## Abstract

**Introduction:**

Persistent postural-perceptual dizziness (PPPD) is a common chronic dizziness disorder with an unclear pathophysiology. It is hypothesized that PPPD may involve functional dysfunction of the construction of inner cognitive maps, leading to disrupted spatial cognition processes as a core feature. The present studies attempt to unravel the neural mechanisms that underlie spatial navigation in PPPD.

**Methods:**

Fifty-two participants completed the study: 19 PPPD patients, 20 control subjects with vestibular disorders but without PPPD (with comparable peripheral vestibular function to the PPPD group, and 13 healthy volunteers). All underwent a virtual Morris Water Maze (vMWM) task in both, non-immersive (NI) and virtual reality (VR) modalities, assessing spatial navigation performance, gaze behavior, and head kinematics.

**Results:**

PPPD patients exhibited significantly worse navigation performance than both control groups across all metrics, with greater impairments in predominantly allocentric tasks. They also showed increased exploratory gaze behavior, unaffected by NI vs. VR modality or task condition. Head kinematics did not significantly differ between the three groups, though a non-significant trend indicated reduced head movement in both PPPD and vestibular controls. VR intolerance was highest in PPPD patients, followed by vestibular controls, with healthy volunteers showing the lowest discomfort.

**Discussion:**

Our findings suggest that PPPD involves deficits in allocentric spatial navigation, likely due to predictive coding errors and impaired internal model updating, rather than sensory input dysfunction. Increased gaze scanning may reflect compensatory mechanisms for spatial uncertainty. Notably, VR immersion did not alter navigation performance, suggesting visuo-vestibular conflict is not the primary driver of PPPD-related spatial deficits. These findings offer new insights into PPPD as a disorder of spatial cognition, opening avenues for novel diagnostic and therapeutic approaches.

## 1 Introduction

Persistent postural-perceptual dizziness (PPPD) is the leading cause of chronic vestibular syndrome, characterized by constant non-vertiginous dizziness, unsteadiness, and sensations of swaying or rocking (1). Despite its prevalence, the underlying pathological mechanisms of PPPD remain unclear, making it a focus of active investigation (2).

The most current pathophysiological model proposes that a triggering event—commonly another vestibular disorder but occasionally a non-vestibular condition such as an acute anxiety episode—initiates a cascade of poorly understood functional neural adaptations (3). These adaptations involve alterations in sensory information processing and vestibular responses within the brain, rather than structural abnormalities (4–7), aligning with the classification of PPPD as a functional neurological disorder (8).

Recent evidence, including work from our group, suggests that these functional neural changes in PPPD may centrally involve higher-order cognitive processes, particularly those related to spatial navigation (9–11). Specifically, we hypothesize that PPPD involves impairments in constructing, updating, and utilizing reliable internal perceptual maps of the external environment and the body’s position within it (10). Errors in predicted perceptual maps and conflicts with re-afferent sensory input from the actual environment are proposed as central mechanisms underlying the core clinical and cognitive phenomena observed in PPPD. We propose that these impairments ultimately give rise to the characteristic symptoms of PPPD and the broader cognitive dysfunctions associated with the condition (including deficits in visuospatial planning, executive function and spatial anxiety among others) (9).

Our previous studies used a virtual Morris Water Maze (vMWM) test in a non-immersive environment to investigate spatial navigation deficits in PPPD patients (9, 10). Since visuo-vestibular conflicts and sensory overload have been proposed as mechanisms underlying PPPD symptoms, navigation impairment could stem not from navigation deficits per se, but from dysfunctions in visual-vestibular processing. To explore this, the present study examines PPPD patient behavior in both non-immersive and immersive virtual reality environments, the latter being more demanding of visual motion processing, as suggested by virtual reality motion sickness studies (12).

Spatial navigation relies on two main strategies. Egocentric navigation is body-centered, encoding locations relative to the individual’s position (e.g., “to my left”) and depends on vestibular, proprioceptive, and motor cues. In contrast, allocentric navigation uses a map-like representation, encoding spatial relationships independently of the individual’s position (e.g., “the building is north of the park”), relying primarily on visual landmarks and cognitive mapping.

In this study, we also aim to determine whether PPPD-related spatial navigation dysfunction is driven primarily by egocentric (vestibular-dependent) processing deficits or by allocentric, visually dependent mapping impairments and how these disruptions affect the reliability of spatial environment predictions. The implications of these findings for the pathophysiology of PPPD will be discussed.

## 2 Materials and methods

A cross-sectional prospective study involving three age-matched groups of subjects: (i) patients diagnosed with PPPD; (ii) patients diagnosed with vestibular disorders other than PPPD; and (iii) healthy volunteers was carried at the neurotology–otolaryngology unit of Clínica Alemana de Santiago in Chile, recruiting attending patients from January 2022 to September 2023. The study was conducted in alignment with the Helsinki declaration, the research received approval from our center’s Ethical Committee (Approval number UIEC 1081). All participants provided written informed consent. Eligibility criteria required participants to be between 18 and 65 years.

The non-PPPD vestibular disorders encompassed benign paroxysmal positional vertigo (BPPV), vestibular neuritis, vestibular migraine (VM), and Ménière’s disease. These disorders were selected because they represent the most common non-PPPD conditions in neuro-otology and result in various types of vestibular dysfunctions. BPPV patients were evaluated before undergoing repositioning maneuvers; all had a history of recurrent BPPV and voluntarily chose to delay treatment to support the study and undergo a complete vestibular assessment. Ménière’s and migraine patients were assessed during inter-ictal periods. Patients diagnosed with vestibular neuritis were only included if they were assessed at least 3 months post-onset, displayed no spontaneous nystagmus, and had not started vestibular rehabilitation by the time of the study procedures.

We recognize the diagnostic intricacies arising from the symptomatic intersection of VM and PPPD. As discussed by Tarnutzer (13), the differentiation between the two is clinically challenging, particularly in cases where a chronic form of VM is present. In such scenarios, it is recommended to consider a coexisting diagnosis of PPPD whenever symptoms persist for more than 3 months. To accurately represent the clinical spectrum, we included VM in both PPPD and non-PPPD cohorts, mirroring its prevalence and the clinical realities encountered in practice. To distinguish between the two, we employed rigorous criteria primarily based on the temporal pattern of vestibular symptoms. For VM, we mandated the presence of discrete episodes with definitive onsets and cessations, and minimal interictal manifestations, requiring at least half of the episodes to include headache or other cardinal VM symptoms (13). For PPPD, we stipulated continuous symptoms, pervasive throughout most of the day and on most days, clearly segregating any overlaid VM episodes (14).

Initial medical consultations for both PPPD and other vestibular diseases adhered to the 2023 Bárány Society diagnostic criteria for definitive disease diagnoses (1, 14–17). After diagnosis determination, examiners conducting assessments were blind to subjects’ groups.

All patients underwent vestibular testing (VNG, vHIT, VEMP) and brain magnetic resonance imaging at 3 Tesla (presented in this manuscript solely to ensure comparability between groups). The primary assessment of this study involved a series of structured virtual (vMWM) spatial navigation challenges, conducted by all participants. These challenges were performed first in a non-immersive (NI) setting using a computer monitor, and subsequently in an immersive virtual reality (VR) environment using VR goggles. In both settings, gaze data were recorded via eye-tracking, while in the VR environment, head kinematics were additionally captured using the built-in sensors of the VR goggles (technical details of the hardware and software used are explained below).

### 2.1 Spatial navigation test

The vMWM served as our primary tool for assessing spatial navigation capabilities (18). The original paradigm was designed for rodents, allowing them to swim freely in a round pool decorated with visual cues. Within the pool lies a transparent platform, hidden slightly underwater from the rodent. To rest, the rodent must first locate and remember this platform’s position, improving its efficiency in reaching it in subsequent trials. Memory impairments, such as from hippocampal lesions, cause rodents to fail in locating the platform. Adapted virtual versions of this test for humans have been validated to identify memory deficits, including those seen in Alzheimer’s patients (19–22).

The vMWM has also been widely used to assess spatial navigation abilities in individuals with vestibular disorders, such as bilateral vestibulopathy (21–23). Our group previously implemented the vMWM, revealing a pronounced and distinct impairment in spatial navigation skills among PPPD patients (9, 10).

For the NI setting, the vMWM was conducted 1.5 m apart from a 24.5-inch desktop monitor. For the VR setting, participants used a the HTC Vive Cosmos, a PC-powered virtual reality headset featuring dual 3.4-inch LCD screens with a combined resolution of 2,880 × 1,700 pixels (1,440 × 1,700 per eye), a 90 Hz refresh rate, and a 110-degree field of view.

For both NI and VR settings participants navigated a virtual environment using a joystick, facilitated by Simian Labs-Maze Engineers®’ Morris Water Maze Software (Build 20,210,821), working on an MSI GT75 Titan computer with a 9SG Intel i9-9980 processor, 64 GB RAM (2,666 MHz) and an NVIDIA RTX 2080 graphics card (8 GB RAM GDDR6), Micro-Star Int’l Co, New Taipei City, Taiwan. This virtual environment comprised a square room (1 × 1 virtual distance units in both “north–south” and “east–west” dimensions) with visual cues centrally positioned on all four walls. At the room’s center was a round pool of 1 virtual unit in diameter.

Participants completed a structured series of six spatial navigation blocks using a virtual Morris Water Maze (vMWM) protocol, applied in both NI and VR environments. The full sequence of blocks was designed to assess training effects, baseline performance, the role of visual cue complexity, and motor control reliability. Specifically, Blocks A, B, and F served to ensure the reliability and internal validity of the protocol, helping to detect potential motor or interface-related confounds and to control for fatigue-related performance changes. To avoid excessive length in the main manuscript, detailed descriptions of each block and their objectives are provided in Supplementary material.

For the purposes of this study, analyses focus on Block C (*“Ego-Allocentric Setting”*) and Block E (*“Mainly Allocentric Setting”*). Both blocks include hidden platform search trials using consistent visual cues, but differ in navigational demands: Block C involves a fixed starting location that permits the use of either egocentric or allocentric strategies, whereas Block E uses randomized starting points and orientations, limiting egocentric guidance and emphasizing allocentric navigation. This contrast allows for targeted evaluation of the spatial navigation mechanisms most relevant to PPPD pathophysiology (10). Various metrics can assess navigational performance in the Morris Water Maze (MWM) paradigm (24–27). In this study, the following parameters were computed to evaluate spatial navigation:

*Path Length*: The total distance (measured in virtual units equivalent to 1 Morris water maze pool diameter) traveled by the participant during each trial, calculated as the sum of the point-to-point distances between consecutive positions recorded during navigation.

*Efficiency Index*: This metric evaluates navigational efficiency by comparing the actual path length to the shortest possible path length required to reach the target. A higher Efficiency Index indicates suboptimal navigation, while a lower value reflects greater proximity to the ideal trajectory.

*Latency:* The time taken by the participant to reach the hidden target during each trial, measured in seconds.

*Cumulative Search Error (CSE):* Also known as Gallagher’s proximity, it represents the average distance between the subject and the target at every timepoint during the trial (24). This metric highlights the efficiency of the search strategy, indicating whether the subject navigates closer to or further away from the target, even if the hidden platform is not directly located.

*Entropy (H)*: Entropy, conventionally denoted by “H,” is a measure of the disorder within a system, reflecting the level of uncertainty or variability in spatial navigation performance. Over the course of training, participants typically transition from a disordered state, characterized by high entropy (e.g., broad and variable search patterns), to an ordered state, characterized by low entropy (e.g., focused searches centered on the former platform location with minimal variance). This shift in search strategy reflects a refinement of navigational behavior.

Entropy measures are particularly useful for understanding the cognitive processes underlying navigation, as they highlight deviations from systematic strategies and quantify the degree of randomness in spatial exploration. In this study, three entropy-related measures were analyzed (26):

*H-error*: Represents the variance in the subject’s position relative to the target. This measure provides insight into the participant’s inconsistency in efficiently reaching the goal. While H-error reflects spatial variability in the subject’s approach to the target, it is conceptually related to CSE, as both parameters capture the efficiency of navigation. However, H-error emphasizes the variability of positional accuracy, whereas CSE integrates both spatial and temporal components into a single efficiency metric.
*H-path*: Represents the variance in the subject’s position with respect to the trajectory itself, independent of proximity to the target. It captures the unpredictability or randomness of the participant’s path, reflecting the complexity and exploratory nature of navigation. Unlike H-error, which focuses on positional accuracy relative to the target, H-path emphasizes the structure and regularity of the trajectory itself. This measure provides additional insight into navigational strategies, as a higher H-path indicates a more erratic or exploratory trajectory, while a lower H-path suggests a more systematic and direct approach. In contrast to CSE, which integrates efficiency in spatial and temporal domains, H-path isolates the randomness inherent in the movement pattern, offering a complementary perspective on navigational behavior.
*H-total*: Given that entropy is additive, combining H-error (navigation error variability) and H-path (trajectory unpredictability) provides a comprehensive measure of navigational behavior. This metric captures both the participant’s accuracy in reaching the target and the degree of randomness or complexity in their exploratory process. By integrating these two components, H-total offers a holistic view of navigational performance, balancing positional precision with the organization (or lack thereof) in the trajectory. Unlike its individual components, H-total reflects the overall cognitive demands and strategies employed during navigation, making it a particularly valuable parameter for understanding global patterns of behavior across different contexts.

Given the differences between the non-immersive (NI) and immersive virtual reality (VR) settings—primarily due to the perception of movement in the maze—and potential variations between experimental blocks (e.g., slightly different distances between start points and hidden targets), we chose to normalize the outputs of each variable within each combination of modality (NI/VR) and experimental blocks. This normalization, expressed as Z-scores, eliminates underlying confounding variables and enhances the reliability of comparisons across settings and contexts. Consequently, most of our data are presented in standardized (Z-score) form rather than in their raw values.

### 2.2 Virtual reality tolerance

Considering the risk of virtual-reality-induced motion sickness, particularly in PPPD patients who are highly sensitive to visual motion, we took proactive measures to address potential discomfort. Participants were instructed to immediately cease the assessment if they experienced dizziness and to report any symptoms of discomfort. For each participant, “VR tolerance” was quantified as the proportion of trials completed relative to the total number of trials in our vMWM protocol (31 trials per NI and VR modalities).

### 2.3 Eye-tracking

Gaze behavior was recorded using Pupil Labs eye-tracking equipment. For the NI setting, the Pupil Labs Core Eye-Tracker was used by all participants throughout the entire vMWM spatial navigation testing. This device consists of a lightweight (22.75 g) headset equipped with a frontal “world camera” (720p at 60 Hz) and two infrared “eye cameras” (192 × 192 px at 200 Hz each), enabling precise tracking of gaze behavior. AprilTag markers, widely used fiducial markers for computer vision tracking, were placed at the corners of the computer monitor where the vMWM experiment was displayed. These markers were used to define the monitor area in the “world camera” recordings as a surface and region of interest (ROI) for gaze analysis. All analyses described herein were conducted using gaze data projected within this defined surface, with the bottom-left corner of the monitor assigned coordinates [0,0] and the top-right corner assigned coordinates [1,1]. From the participant’s perspective, the horizon of the navigating pool was aligned with the middle of the screen (0.5 on the y-axis of the monitor). Coordinates projected above the 0.5 y-axis on the screen included elements of the virtual environment such as the round pool wall, the outer square walls, and, most importantly for data interpretation, the pictures used as visual cues. In contrast, coordinates at or below the 0.5 midline represented the water surface within the experiment, as well as the red goal target when it was visible.

For the VR setting, the Pupil Labs HTC Cosmos VR Add-on was implemented and mounted on the HTC Vive Cosmos Virtual Reality headset. This device includes two infrared “eye cameras” (192 × 192 px at 200 Hz each), integrated into a slim ring adapter designed specifically for eye movement tracking during immersive virtual reality use. Given the immersive nature of the VR setting, it is not meaningful to define the borders of the “screen” or virtual experience, as the visual coordinates extend beyond the [0–1] range. We carefully calibrated the headset’s coordinate system to ensure that the 0.5 y-axis midline aligned with the horizon of the vMWM environment. Additionally, when determining gaze coordinates, we applied corrections for shifts in head orientation caused by head rotation.

Recordings for both settings were acquired using Pupil Labs Capture Software (v3.3.0), ensuring consistency in data collection and compatibility with subsequent analysis pipelines. The main metrics analyzed in this study were fixations and scanned path. Fixations were characterized by their position, frequency, and duration on the visually projected surface, calculated using the default parameters provided by Pupil Labs Software. These metrics were intended to reflect areas of visual interest.

Scanned path was calculated as the cumulative distance between consecutive gaze points, encompassing saccades, microsaccades, and other eye movements. This metric was used to quantify the extent of visual exploration within the virtual environment, providing insights into participants’ navigational and exploratory strategies.

### 2.4 Head kinematics

Head kinematics were recorded using the internal accelerometers of the HTC Vive Cosmos Virtual Reality Headset, which provides six degrees of freedom. Rotational movement was measured in the yaw, pitch, and roll axes (degrees), with the default position aligned to the horizon of the pool in the vMWM setting. Translational movement along all axes was minimal and did not differ significantly across groups or experimental settings; therefore, this data is not presented in this manuscript.

### 2.5 Vestibular and imaging testing

To support comparability across groups and to rule out central neurological conditions that could confound spatial navigation results, all participants underwent a comprehensive vestibular evaluation—including video head impulse testing (vHIT), vestibular evoked myogenic potentials (VEMPs), and videonystagmography (VNG)—as well as brain magnetic resonance imaging (MRI). These procedures were conducted to confirm peripheral vestibular function and exclude structural abnormalities in regions relevant to spatial cognition. Full methodological details of these assessments are provided in Supplementary material.

## 3 Results

Sixty-three individuals who met the inclusion criteria were invited to participate. Of these, 11 declined, while 52 agreed and completed all assessments. Nineteen patients who met the criteria for PPPD were included in the “PPPD” group. Twenty patients, though not meeting the PPPD criteria, were diagnosed with other vestibular disorders and were placed in the “vestibular” control group. The conditions BPPV, Ménière’s disease, vestibular migraine, and acute vestibulopathy were comparably distributed between the two groups. A separate “control” group consisted of 13 healthy volunteers. The primary characteristics of all participants are summarized in Table 1. Importantly and as intended, no significant difference was found on age [ANOVA *F*_(2,49)_ = 0.253; *p* = 0.73] or educational level [ANOVA *F*_(2,49)_ = 0.324; *p* = 0.72] between groups (given the known influence of these factors over cognitive performance). The neuroradiological evaluation of magnetic resonance brain scans yielded no abnormalities. Specifically, no hippocampal lesions were identified.

**Table 1:** Demographic summary of PPPD, vestibular, and control groups.

Variable		Group		
		PPPD n=19	Vestibular (Non-PPPD) n=20	Healthy control n=13
Age^*^	Mean	46.8	44	43.8
	Standard deviation	14.9	13.9	15.2
	Range	21–65	20–63	25–64
Gender	Female/Male	79%/21%	85%/15%	77%/23%
Educational level^*,†^	Mean score	3.78	3.9	3.76
	Standard deviation	0.53	0.31	0.41
Diagnosis (percentage of each group)	Vestibular migraine	21.6%	33.3%	-
	Vestibular neuritis	24.3%	23.3%	-
	Benign positional paroxysmal vertigo	10.8%	23.3%	-
	Bilateral vestibulopathy	2.7%	10%	-
	Otoesclerosis	2.7%	3.3%	-
	Meniere’s disease	2.7%	3.3%	-

^*^Non-significant differences were found on age (ANOVA F = 0.264; *p* = 0.76) or educational level (ANOVA F = 0.307; *p* = 0.73) between groups. ^†^Educational level was classified as 1 = primary education incomplete, 2 = primary education complete, 3 = secondary education complete, 4 = undergraduate education complete, 5 = postgraduate education complete.

To assess group comparability, we rigorously analyzed vestibular function. The PPPD and vestibular groups demonstrated significantly reduced vestibular function compared to healthy controls, as determined by vHIT and VEMP assessments (ANOVA with Tukey *post hoc*, *p* < 0.05). Despite this reduction, there was no significant difference between the PPPD and vestibular groups, suggesting similar levels of dysfunction. Supplementary Table S1 contains comprehensive results, including mean VOR gain, the proportion of patients with gains below 0.7, corrective saccades, and VEMP response amplitudes, along with detailed statistical analyses for each variable.

Our results are presented in four main sections. First, we report differences in spatial navigation performance across groups and experimental conditions, with special focus on allocentric navigation and entropy-based metrics. Second, we analyze gaze behavior, including fixation patterns and overall scene exploration. Third, we present head kinematics during immersive navigation. Finally, we report tolerance to the virtual reality environment across groups.

### 3.1 Spatial navigation results

#### 3.1.1 PPPD participants perform worse navigationally in all metrics

**Figure 1:**
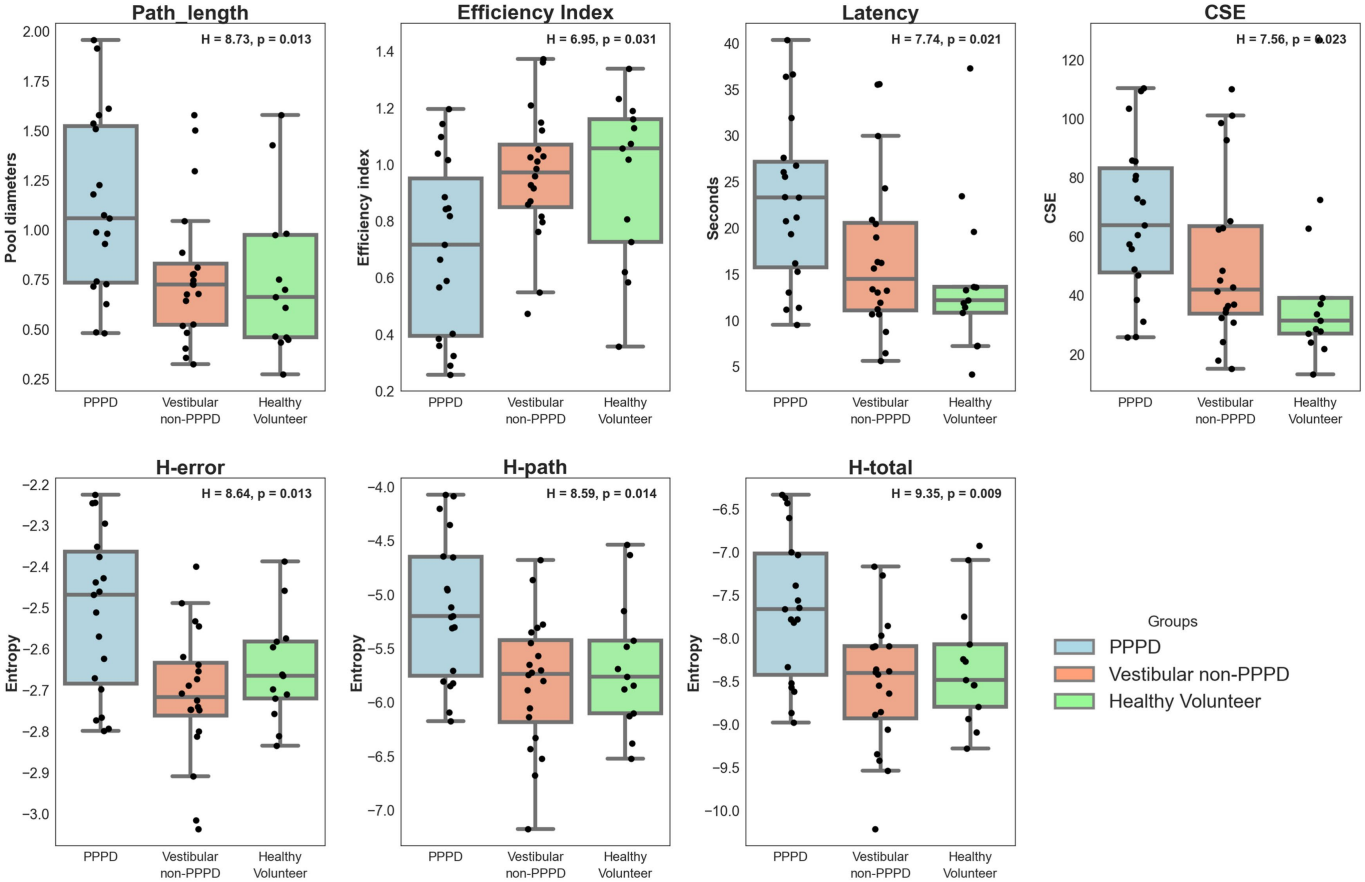
Overall spatial navigation performance. Boxplots illustrating the spatial navigation performance metrics analyzed in this study, comparing the PPPD group with both control groups. Results are presented for the combined performance in the blocks of greatest interest: the Ego-Allocentric and Mainly Allocentric settings. Metrics are shown for both the non-immersive (NI) and virtual reality (VR) contexts. For each metric, the Kruskal-Wallis “H” statistic and corresponding *p*-value are reported. Note that for all metrics, except the Efficiency Index, larger values indicate poorer navigation performance.

Due to heterogeneous variances (Levene’s Test: W = 4.185, *p* = 0.021), non-parametric tests were used for analysis. Figure 1 provides an initial overview of spatial navigation performance, combining results from all navigation trials in the blocks of greatest interest (Ego-Allocentric and Mainly Allocentric settings) across both non-immersive (NI) and virtual reality (VR) contexts (Larger values for all metrics indicate worse navigation performance, except for the Efficiency Index, where larger values reflect better navigation).

Significant differences were observed between groups across all metrics, with Kruskal-Wallis tests yielding *p*-values less than 0.05 (detailed H and *p*-values annotated in each sub-plot in Figure 1). *Post-hoc* Dunn’s tests revealed that for every metric PPPD participants exhibited worse spatial navigation performance compared to at least one of the control groups.

The strongest statistical significance, including consistent differences between PPPD patients and both control groups in post-hoc testing, was observed for entropy measurements, with H-total showing the highest significance. Additionally, CSE was the only metric where a Dunn’s test (*p* < 0.005) also revealed differences between the two control groups, with vestibular non-PPPD patients performing worse than healthy volunteers.

Considering the apparently stronger sensitivity of entropy (with lower *p*-values) and the differentiating potential of CSE (Identifying a difference in performance between vestibular-non PPPD and Healthy Volunteers), further analyses were focused on these two metrics.

#### 3.1.2 There is no difference in navigation across non-immersive and virtual reality modalities

**Figure 2:**
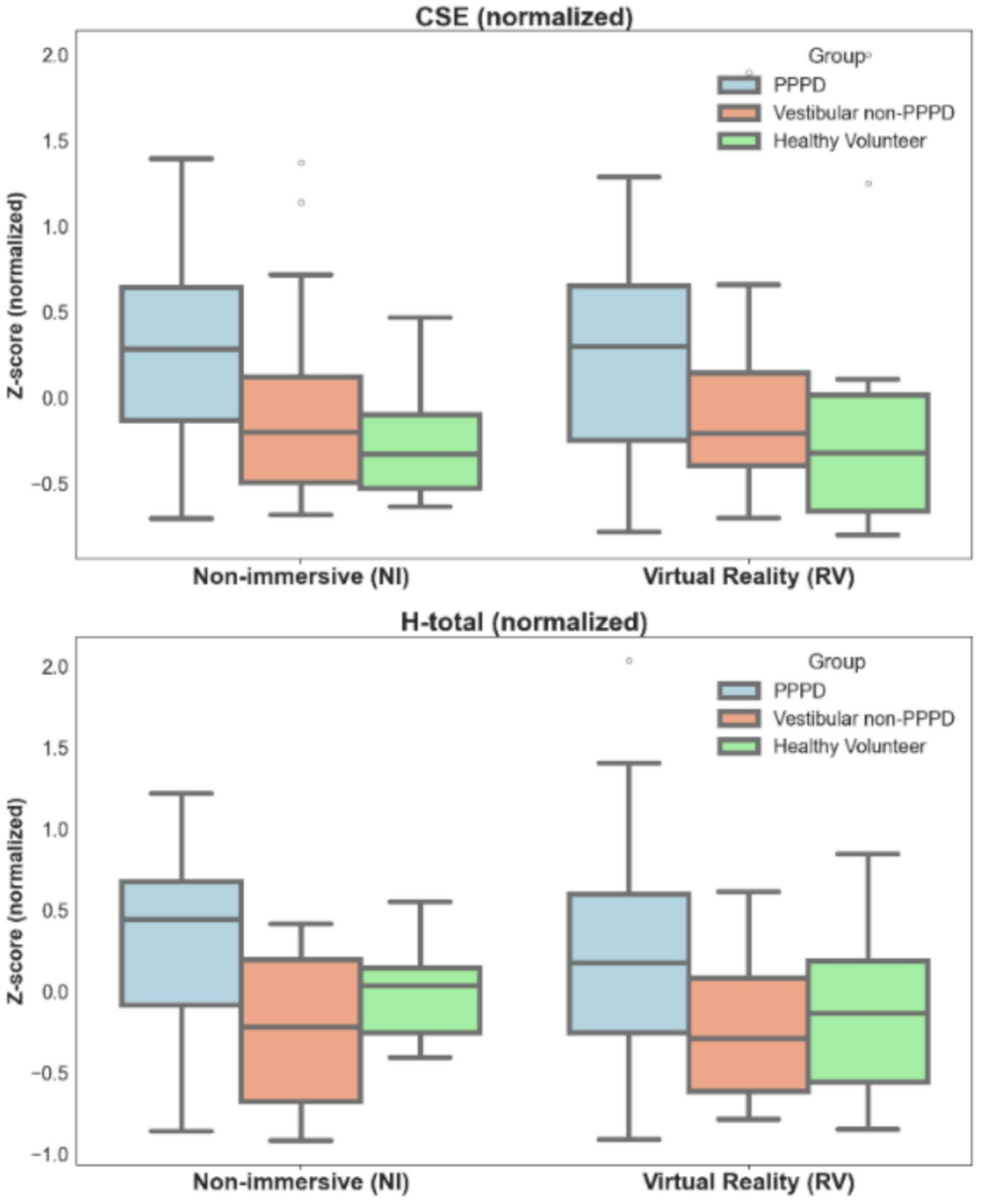
Differences in spatial navigation performance across modalities (NI-VR). Boxplots illustrating spatial navigation performance metrics in terms of both CSE and H-total across non-immersive (NI) and virtual reality (VR) modalities. Metrics are presented in normalized values to allow for appropriate comparisons. Differences between groups (PPPD and controls) are evident, with no significant changes observed across modalities (see text for statistical details).

Figure 2 illustrates whether spatial navigation performance differs when conducting the vMWM challenge in NI or VR modalities. For both metrics explored (CSE and H-total), no significant differences were found using Kruskal-Wallis tests when comparing navigational performance within groups across modalities (e.g., PPPD patients did not perform better or worse in VR compared to NI, and the same was true for vestibular non-PPPD and healthy controls).

The differences in performance between PPPD patients and both control groups observed in Figure 1 remained consistent across modalities. For CSE, Kruskal-Wallis values and *p*-values ranged from H = 6.123 to H = 3.132 and *p* = 0.039 to *p* = 0.047, with post-hoc tests indicating significant differences across all group combinations (PPPD > Vestibular > Healthy Controls). For H-total, Kruskal-Wallis values and p-values ranged from H = 9.288 to H = 4.202 and *p* = 0.010 to *p* = 0.029, with post-hoc tests showing significant differences only between PPPD patients and both control groups.

To explore whether modality (using VR instead of NI) exacerbates or diminishes intergroup differences, we conducted a bootstrap test to compare the effect sizes of these differences across modalities. For CSE, we obtained Δη^2^ = −0.042, Confidence Interval (CI): [−0.291, 0.211]; for H-total, Δη^2^ = −0.084, CI: [−0.375, 0.209]. These results indicate that modality had no significant effect on the intergroup differences in spatial navigation performance.

#### 3.1.3 PPPD impairment is exacerbated in a mainly allocentric setting

**Figure 3:**
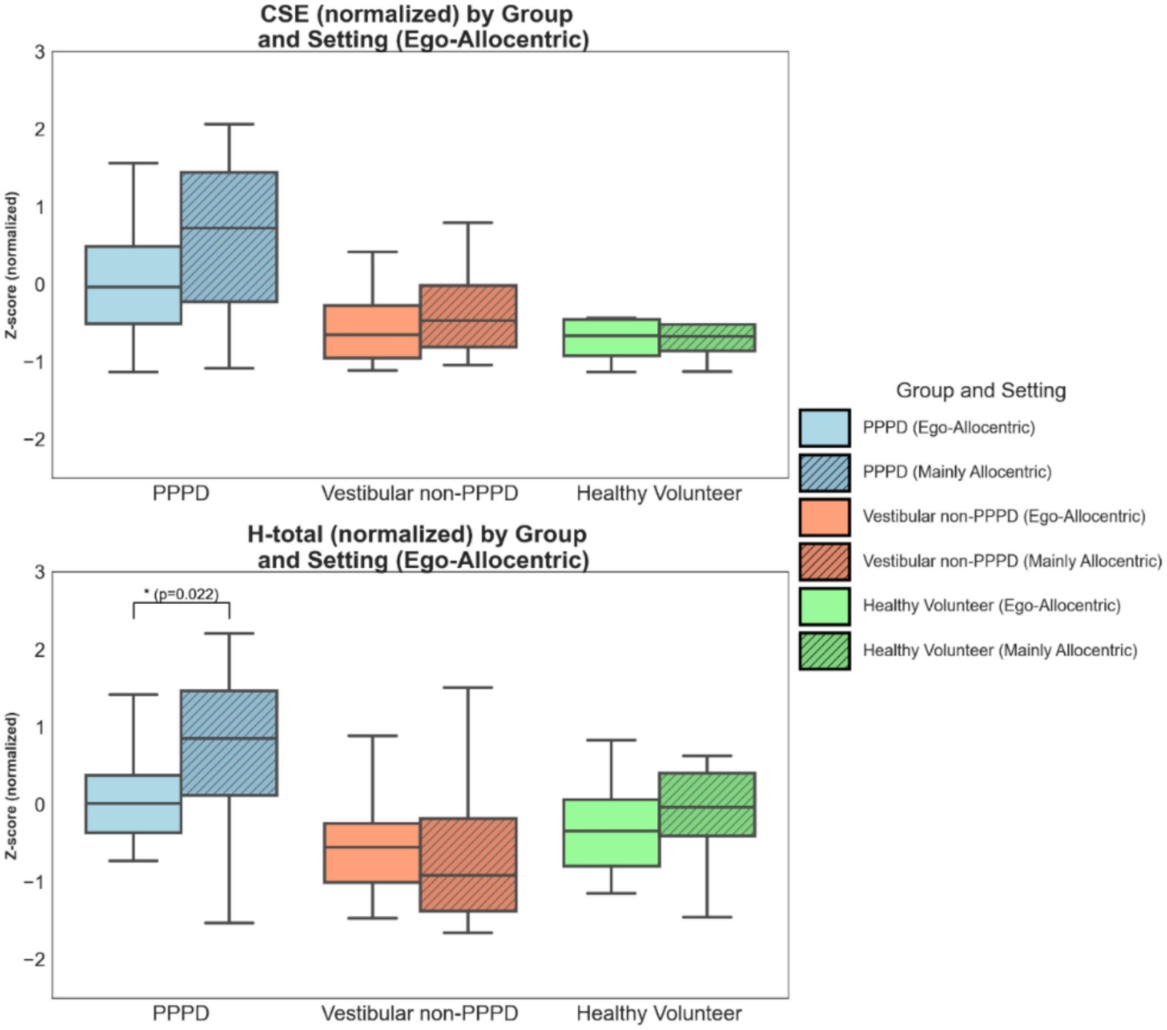
Differences in spatial navigation performance across settings (favored navigational strategy: Ego- vs Allocentric). Boxplots illustrating spatial navigation performance metrics (CSE and H-total) between two settings of the vMWM challenge: one with a fixed starting point and orientation (where both egocentric and allocentric cues are available) and another with a random starting point and orientation (where allocentric cues are predominantly required). Metrics are presented in normalized values to facilitate appropriate comparisons. The effect size of intergroup differences is greater in the Mainly Allocentric setting (see text for statistical details). For H-total, PPPD participants exhibited significantly worse navigation performance in the Mainly Allocentric setting in Kruskal-Wallis test (H = 5.252; *p* = 0.022).

Figure 3 presents the differences in CSE and H-total across the three groups in two key blocks of the vMWM protocol. Block C, with a fixed starting point and orientation, provides both egocentric and allocentric cues for navigation. In contrast, Block E, with random starting points and orientations, primarily tests allocentric navigation skills required to locate the hidden target.

For both metrics, CSE and H-total, and consistent with previous results, significant differences were observed between clinical groups in both the Ego-Allocentric and Mainly Allocentric settings (Kruskal-Wallis H values ranged from 16.144 to 6.545; *p*-values ranged from 0.001 to 0.041). Post-hoc Dunn’s tests revealed that PPPD participants performed significantly worse than both control groups for both metrics.

In a bootstrap analysis similar to the one performed for the NI vs. VR comparison, we found that the size effect of intergroup differences was larger in the Mainly Allocentric setting. Specifically, the “worsening” of performance associated with being part of the PPPD group was more pronounced when primarily allocentric cues were required: CSE Δη^2^ = 0.284, CI [0.310, 0.909]; H-total Δη^2^ = 0.321, CI [0.519, 1.106].

Additionally, within the PPPD group, a significantly worse navigational performance was observed in the Mainly Allocentric setting compared to the Ego-Allocentric scenario for H-total (Kruskal-Wallis test: H = 5.252, *p* = 0.022).

**Figure 4:**
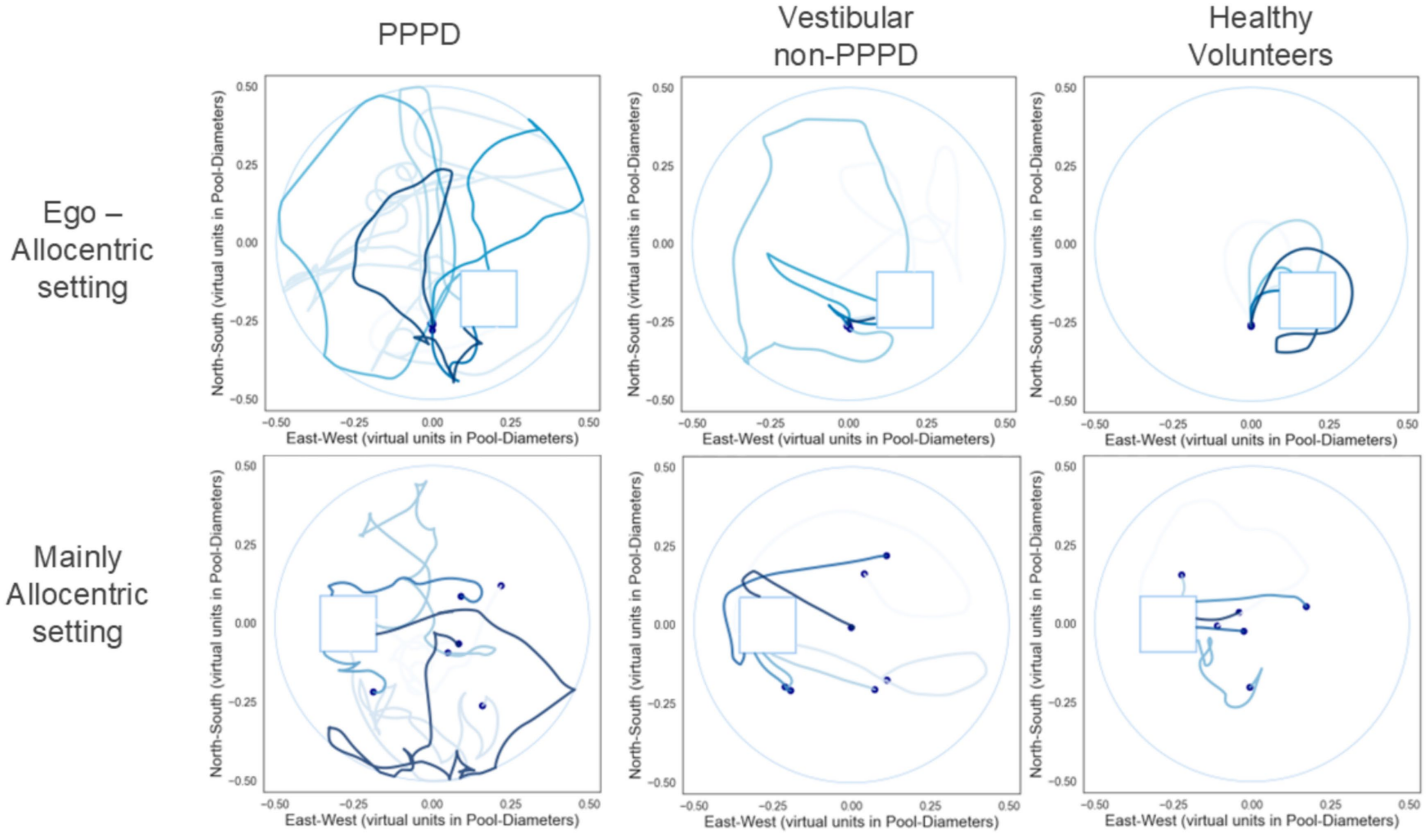
Navigation patterns in vMWM pool. Representative navigation paths from qualitatively selected cases within each group are shown to visually illustrate typical trajectory patterns. The upper row depicts data from the Ego-Allocentric setting, while the lower row shows data from the Mainly Allocentric setting. In each plot, varying shades of blue represent the trajectories from individual trials. The location of the hidden target is marked by a light blue square.

**Figure 5:**
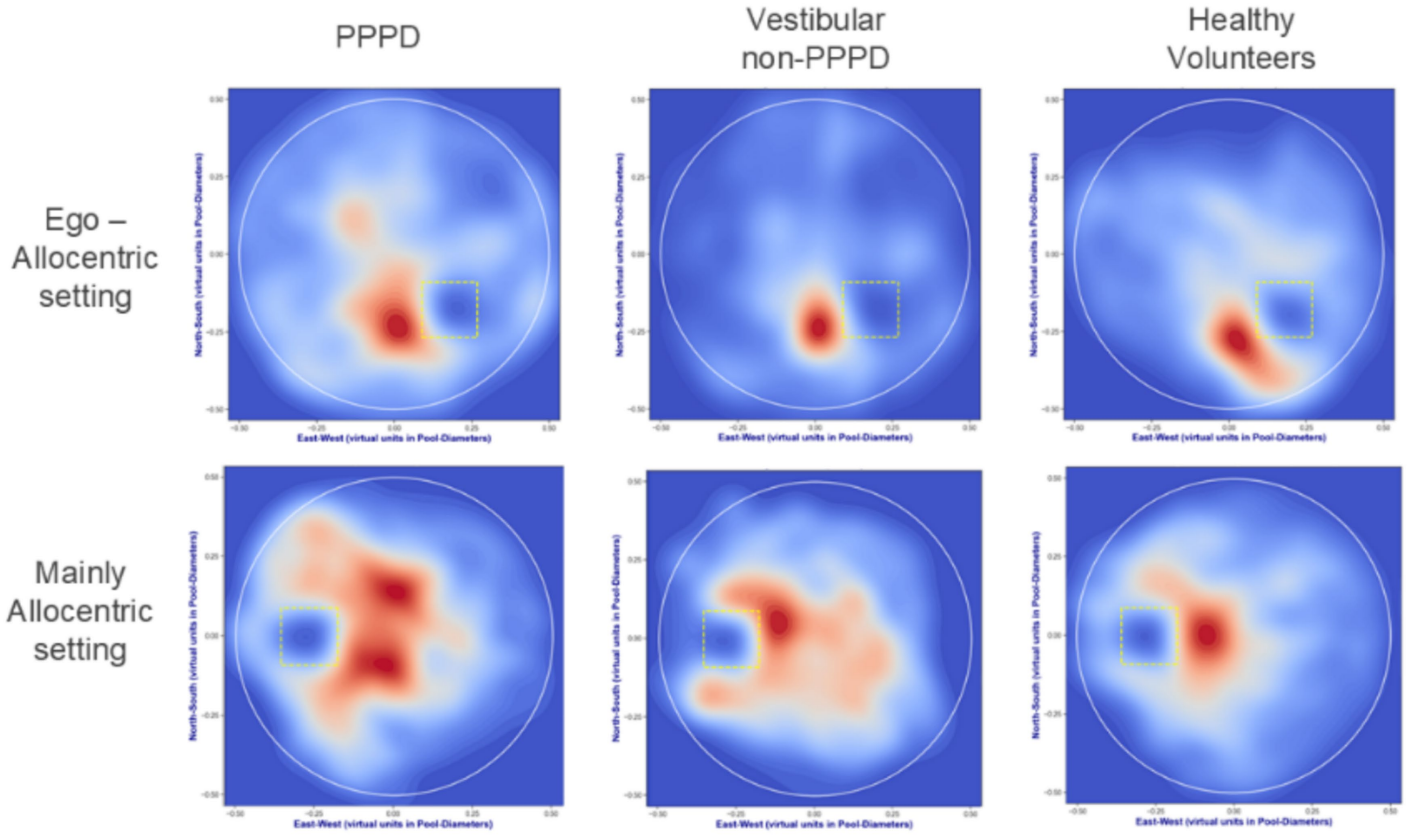
Navigation heatmaps in vMWM pool. Density plots derived from kernel estimations highlight frequently visited areas during navigation in both Ego-Allocentric and Mainly Allocentric contexts. These plots illustrate each group’s overall behavior relative to the hidden target, represented by yellow dotted squares. Red regions denote the most frequently visited areas, while blue regions indicate less visited zones, normalized across 50 probability levels. Healthy controls predominantly navigated close to the hidden target, as evidenced by concentrated red regions near the target and extensive deep blue areas elsewhere, indicating a low likelihood of being found outside the target vicinity. Vestibular non-PPPD patients displayed a broader navigation pattern, though still target-focused. In contrast, PPPD patients, while exhibiting some target-directed behavior, navigated a more expansive range of areas, reflecting a less efficient and more exploratory strategy.

Figures 4, 5 are presented to provide a more qualitative demonstration of the differing navigation performances and strategies observed across groups. Figure 4 displays selected examples of navigation paths from each group, while Figure 5 presents heatmaps summarizing the behavior of all participants within a given group. In general, vestibular patients appeared to adopt navigation strategies similar to those of healthy controls, albeit with less efficiency. In contrast, PPPD patients exhibited significantly more erratic and disorganized navigation patterns, including circular search behaviors within confined areas. This tendency toward disorganization and erratic searching was qualitatively more pronounced in the Mainly Allocentric setting, suggesting a further exacerbation of their navigational difficulties under these conditions.

#### 3.1.4 Entropy analysis suggests a greater impact of PPPD on target position uncertainty, particularly in the mainly allocentric challenge

Since the entropy metrics used in this study capture two different aspects of uncertainty—one related to trajectory uncertainty concerning the hidden target (which could reflect acquired knowledge about its location on the map) and another associated with general trajectory uncertainty (reflecting randomness, exploration variability, or errors in movement within the pool)—it is of interest to determine which type of entropy predominates. To assess this, we calculated an Entropy Ratio, defined as H-error/H-total, where H-total is the sum of H-error (target-related uncertainty) and H-path (trajectory-related uncertainty).

**Figure 6:**
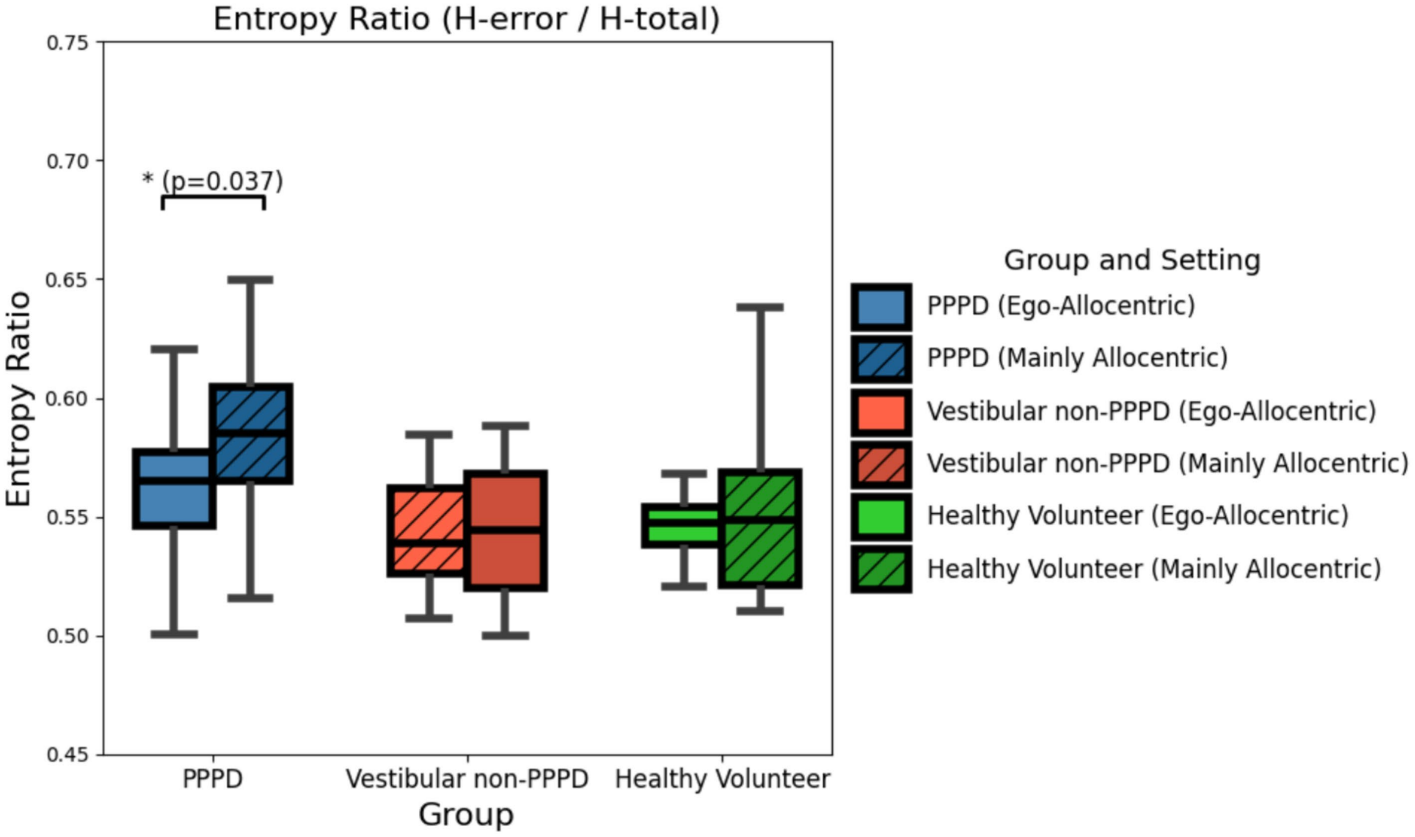
Entropy (H-error/H-total) ratio across groups and settings. Boxplots illustrating the ratio between H-error and H-total (the sum of H-error and H-path) across all groups and in both Ego-Allocentric and Mainly Allocentric settings. In all cases, H-error slightly dominates over H-path, with a ratio of approximately 0.55 in favor of the former. However, this dominance is significantly more pronounced in the PPPD group within the Mainly Allocentric setting (Kruskal-Wallis test: H = 4.357, *p* = 0.037). This finding suggests that, in the PPPD group, the uncertainty in navigation related to knowledge of the target’s position is disproportionately higher than the overall uncertainty of navigation within the vMWM challenge. This effect appears particularly exacerbated when allocentric cues are predominantly required.

As shown in Figure 6, all groups and settings exhibit a slight predominance of H-error, with an entropy ratio slightly above 0.55 (where a ratio of 0.5 would indicate equal contributions from both entropy types). Notably, the PPPD group shows a significantly higher proportion of H-error compared to control groups (Kruskal-Wallis H = 9.669; *p* = 0.007). This finding expands upon and refines the pattern observed in Figure 1, highlighting that, beyond a general increase in entropy, PPPD participants exhibit a disproportionate uncertainty specifically related to the hidden target’s location. Interestingly, this proportion is further amplified in the Mainly Allocentric setting, where it differs significantly from the Ego-Allocentric condition (Kruskal-Wallis test: H = 4.357, *p* = 0.037).

### 3.2 Eye-tracking results

**Figure 7:**
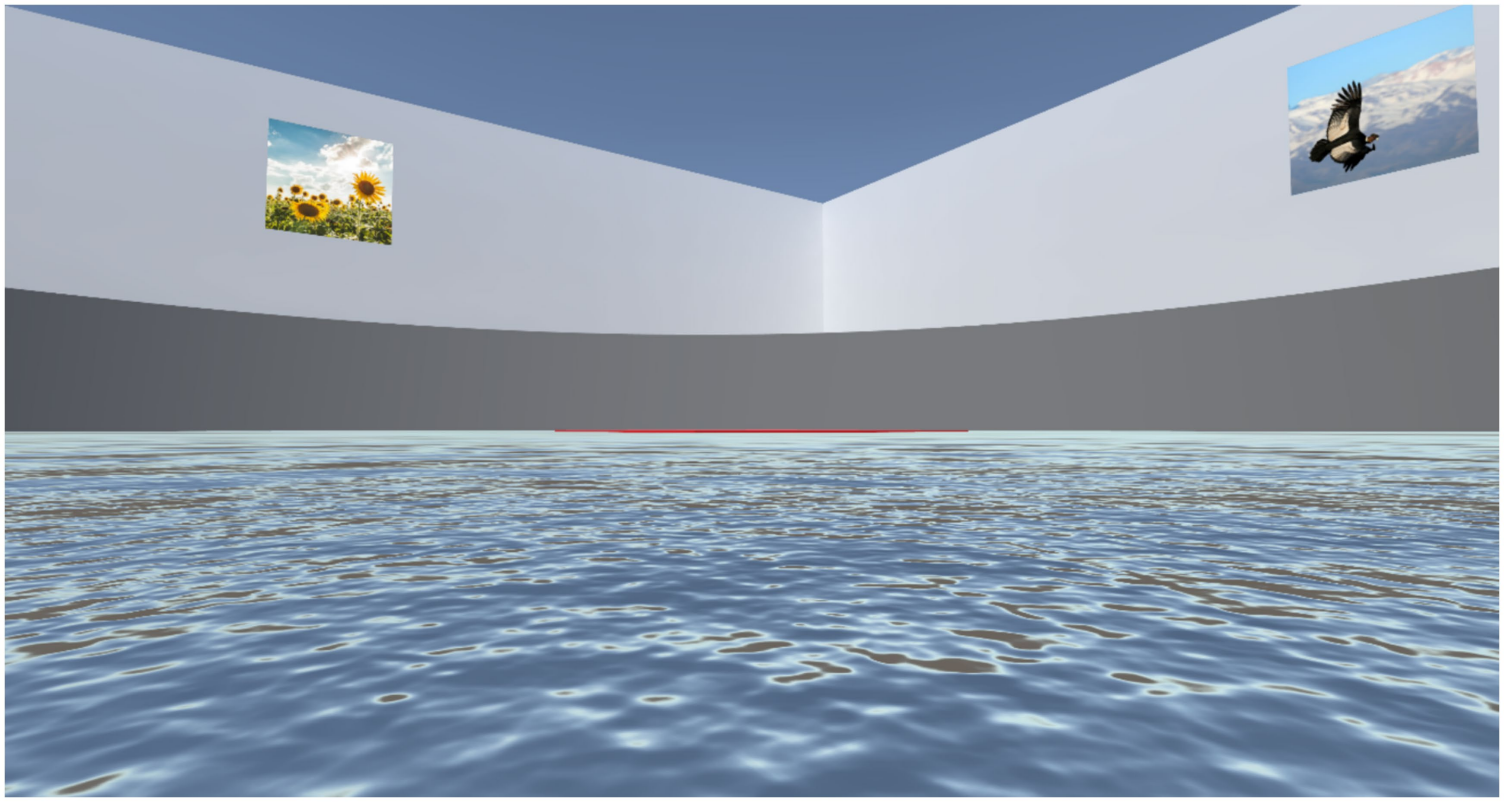
Example of a participant’s view in the vMWM challenge. To facilitate a better understanding of the subsequent gaze distribution figures, this image presents an example of the visual experience encountered by participants during the navigation test. From the bottom of the image upwards, the following elements can be observed: (i) The virtual water of the vMWM pool; (ii) The target, represented by a thin red line from this distant perspective to ensure all elements are visible (note that in both experimental settings, the target remains hidden until found; it is shown here for demonstration purposes); (iii) The round pool wall in dark gray; (iv) The square room’s walls in light gray, enclosing the pool; (v) Two of the four visual cues hanging on the walls of the square room; (vi) The ceiling of the entire scenario, depicted in deep blue.

Gaze behavior over the projected scene of the vMWM challenge was assessed in both NI and VR modalities. To provide a more intuitive interpretation of our findings, Figure 7 presents an example of the actual scene experienced by participants during testing. The screen was normalized using relative coordinates, with [0,0] representing the bottom-left corner and [1,1] the top-right corner.

For the VR modality, due to its immersive nature and the correction of head orientation in eye-tracking calculations, a strictly delimited coordinate system was not appropriate. Instead, for this modality, the area of interest for analysis ranged from 0 to 1 on the x-axis and from −0.5 to 1.5 on the y-axis.

#### 3.2.1 PPPD appears to distribute fixations more broadly over visual cues area

**Figure 8:**
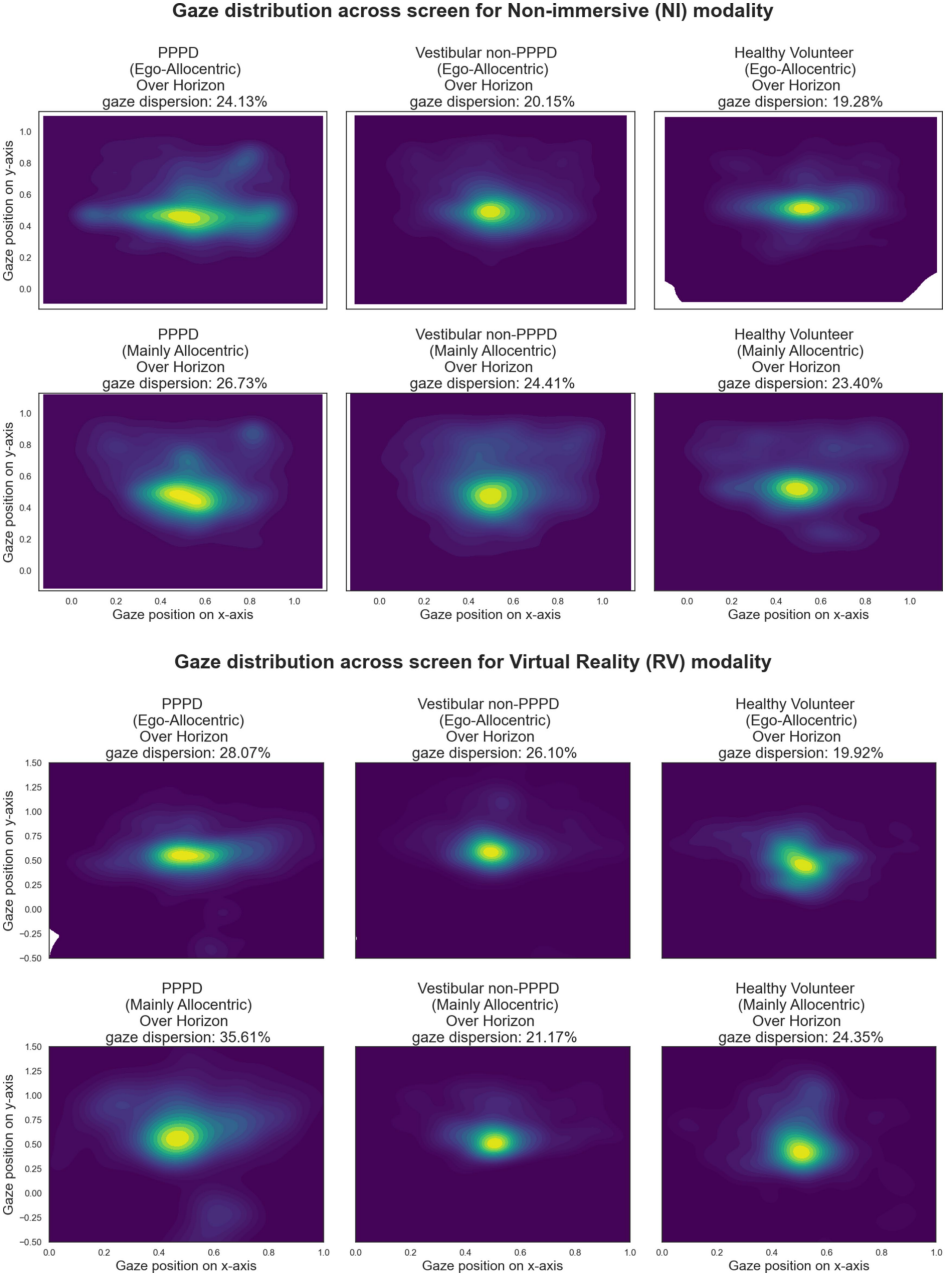
Heatmaps illustrating the spatial distribution of gaze fixations over the vMWM scene for each group-modality-setting. Brighter yellow areas indicate regions with higher fixation density, while darker blue areas represent less frequently fixated zones. Across all conditions, a strong concentration of fixations is observed at the scene’s center and along the y = 0.5 horizon line, where visual cues are located. PPPD participants appear to exhibit a broader fixation distribution over these regions compared to controls. The “Over-Horizon Gaze Dispersion” metric, quantifying the proportion of fixations along the horizon line (excluding central fixations), is reported in each panel. While PPPD participants show numerically higher values, differences between groups were not statistically significant.

Figure 8 presents heatmaps of fixation locations across the vMWM scene (as depicted in Figure 7). Brighter yellow areas indicate regions where gaze fixations were most concentrated, while darker blue zones represent areas with fewer fixations. Heatmaps for each group-modality-setting are shown.

Across all conditions, a strong concentration of fixations is observed at the exact center of the scene—representing the reference point from which motion perception occurs, akin to looking toward the horizon while walking. Notably, this central region also corresponds to the location where the hidden target becomes visible once found. Additionally, in every heatmap, fixations are more broadly distributed along the y = 0.5 horizon line, where the visual cues are placed.

Qualitative inspection suggests that PPPD participants exhibit a broader fixation distribution in these regions compared to both control groups. To quantify this observation, we calculated the percentage of gaze fixations landing along the horizon line, excluding fixations at the exact center of the screen. This was done by removing fixations within a [0.2 × 0.2] square centered at [0.5, 0.5], defining a metric we refer to as “Over-Horizon Gaze Dispersion.” The values for this metric are reported in each corresponding panel of Figure 8.

Although PPPD participants showed numerically larger dispersion values, these differences did not reach statistical significance in formal tests.

#### 3.2.2 PPPD gaze transverses more of the visual scene during navigation

**Figure 9:**
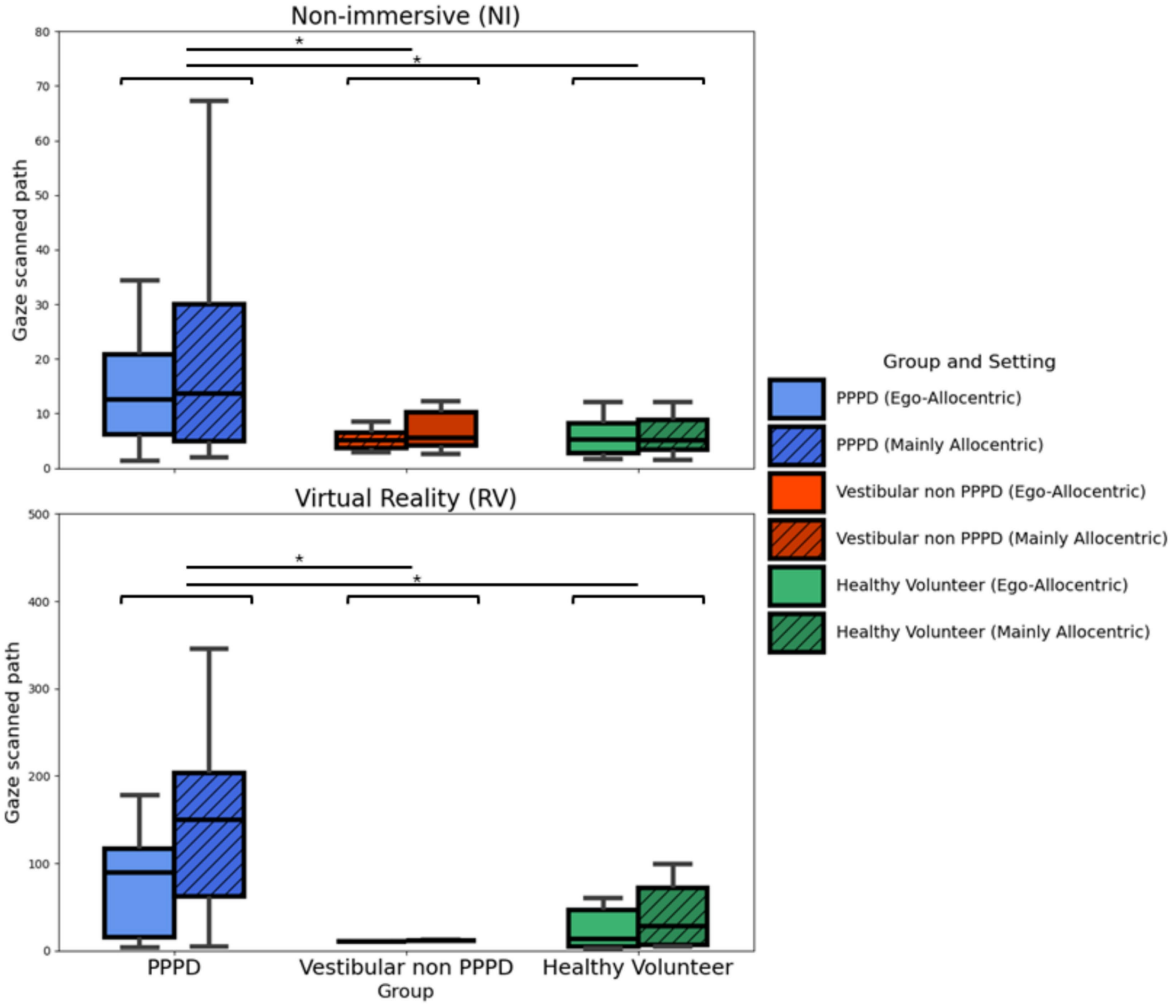
Gaze scanned path across modalities and settings. Boxplots illustrating the total sum of the distances between consecutive gaze points, providing a sensitive measure of gaze dispersion and exploratory behavior during navigation. While no statistically significant differences were found between settings (Ego-Allocentric vs. Mainly Allocentric) or between modalities (NI vs. VR), PPPD patients consistently exhibited significantly greater scanned path distances compared to both control groups (Kruskal-Wallis H = 8.075–6.598; *p* = 0.017–0.040).

A sensitive metric for assessing visual exploration is the total sum of gaze scanned path, calculated as the cumulative distance between consecutive gaze points. This metric encompasses saccades, microsaccades, and all other eye movements performed during the task. Figure 9 summarizes this metric across group-setting-modality conditions. In every scenario, PPPD participants exhibited significantly greater scanned path distances compared to both control groups (Kruskal-Wallis H = 8.075–6.598; *p* = 0.017–0.040). However, no significant differences were observed between Ego-Allocentric settings or between NI and VR modalities.

**Figure 10:**
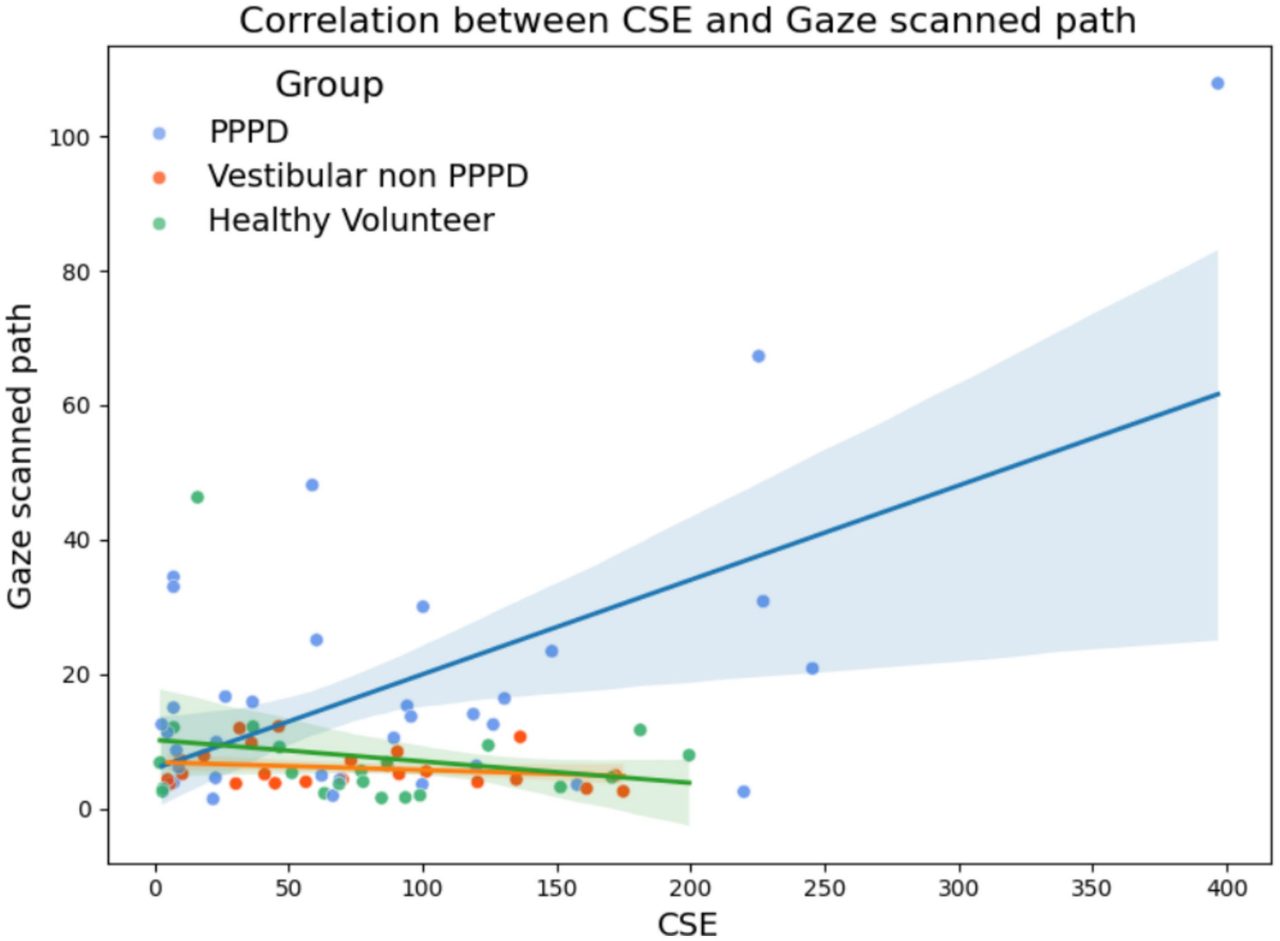
Relationship between gaze scanned path and spatial navigation performance (CSE). Scatter plots illustrating the correlation between gaze scanned path and CSE across groups. While no significant Spearman correlations were found (nor in other spatial navigation performance metrics), a trend suggests that increased gaze scanned path is associated with poorer navigation performance in PPPD participants (Spearman’s Rho = 0.59; *p* = 0.125), whereas this relationship is not observed in either control group.

Given the clear difference in more active exploratory gaze behavior observed in PPPD patients compared to both control groups, we investigated the potential correlation between this metric and spatial navigation performance. Figure 10 illustrates this relationship with CSE, where, although no significant Spearman correlations were found, a trend suggests that a greater scanned path is associated with poorer navigation performance in the PPPD group (Spearman’s Rho = 0.59; *p* = 0.125). Notably, this relationship is absent in both control groups.

### 3.3 PPPD head kinematics results

**Figure 11:**
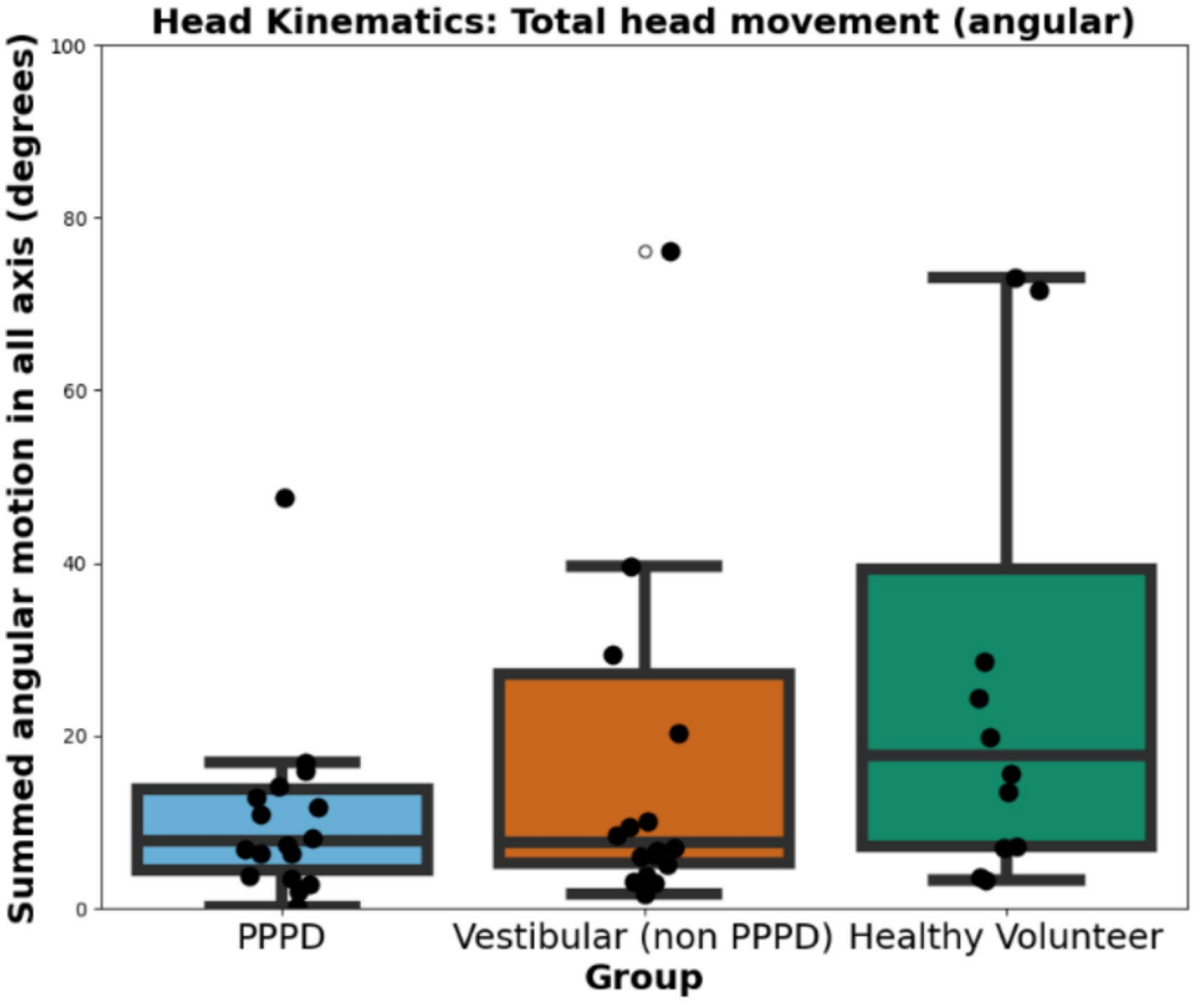
Head movement during immersive navigation. Boxplots illustrating the total sum of rotational changes across all axes (in degrees per minute) during the VR navigation challenge. While no statistically significant differences were found between groups, healthy volunteers exhibited a slight tendency to move their heads more freely compared to PPPD and vestibular non-PPPD participants.

The VR headset used in this study allowed us to assess the extent to which participants relied on head movements to orient themselves during the immersive navigation challenge (which could be a sign of egocentric-cue dependency), rather than solely using gaze direction changes. An integrative measure of this behavior was the total sum of rotational changes across all axes (in degrees) per minute. Figure 11 presents this metric across groups. Statistical analyses revealed no significant differences between groups. However, in terms of trends, healthy volunteers exhibited a slightly greater tendency to move their heads more freely during navigation, possibly reflecting a lack of fear of head movement, which patients with vestibular disorders may experience. Nonetheless, this difference did not reach statistical significance (H = 2.785; *p* = 0.24).

### 3.4 Virtual reality tolerance results

**Figure 12:**
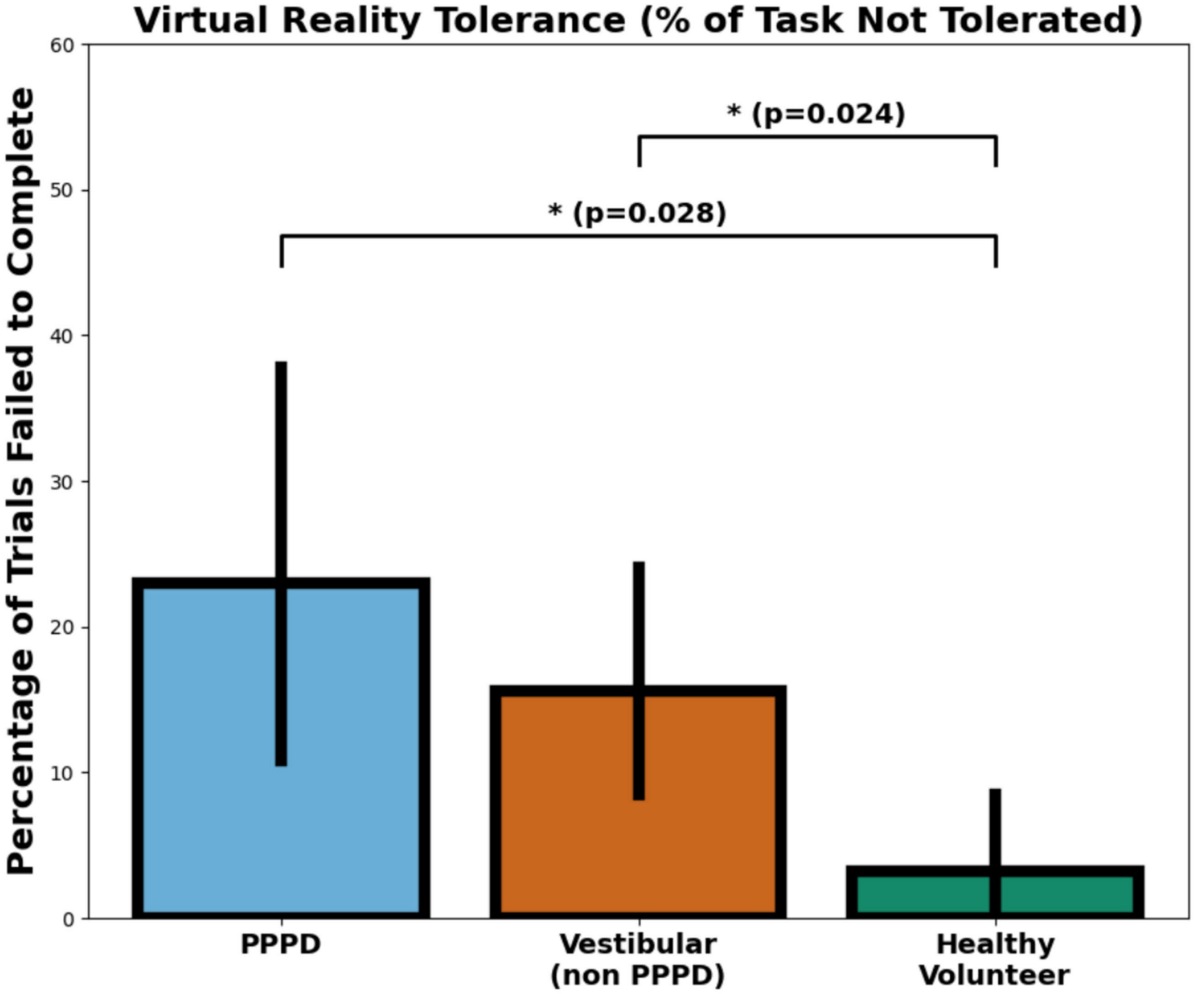
Virtual reality tolerance across groups. Boxplots illustrating the percentage of vMWM trials not completed due to dizziness, nausea, or significant discomfort in the VR modality. PPPD participants exhibited the highest level of intolerance, with a statistically significant difference compared to healthy volunteers (*p* = 0.028). While vestibular non-PPPD participants also showed greater VR intolerance than healthy volunteers, the difference between PPPD and vestibular non-PPPD groups did not reach statistical significance.

To assess tolerance to the vMWM experience, we calculated the percentage of trials that participants were unable to complete due to dizziness, nausea, or significant discomfort. All participants successfully completed the vMWM protocol in the NI setting, so tolerance was assessed only for the VR modality. Figure 12 presents these results, showing that PPPD participants exhibited the highest level of intolerance among all groups. The difference in VR tolerance was statistically significant between PPPD and healthy volunteers (*p* = 0.028), but not significant between PPPD and vestibular non-PPPD participants. Additionally, vestibular non-PPPD participants showed significantly greater VR intolerance than healthy volunteers.

From a qualitative perspective, researchers observed that within the vestibular non-PPPD group, those with greater VR intolerance tended to have a stronger history of vestibular migraine (also, 62.5% of individuals in this group who failed more than 10% of trials presented vestibular migraine, and all subjects failing more than 20% of trials has vestibular migraine). However, as previously noted, the prevalence of vestibular migraine was the same in both the PPPD and vestibular non-PPPD groups.

## 4 Discussion

### 4.1 PPPD as a dysfunction of the predictive processing

PPPD represents a challenge in clinical practice—given the difficulties in properly identifying and treating many patients—as well as from an epidemiological and healthcare system perspective, as it ranks among the three most common causes of dizziness (2). Additionally, from a neuroscientific and mechanistic standpoint, the pathophysiological mechanisms underlying PPPD remain a topic of ongoing debate (2, 3, 8, 28, 29).

The currently accepted and clinically useful explanatory model of PPPD focuses on behavioral changes observed in affected individuals, such as increased body vigilance, heightened visual dependence, and stiffened postural control (3). However, given that PPPD is classified as a functional neurological disorder, a fundamental question arises: Is there a broader, higher-order dysfunction underlying the mechanisms of PPPD, from which these behavioral alterations emerge as consequences rather than as the primary issue? This perspective aligns with contemporary frameworks used to explain functional disorders, in which multiple pathophysiological factors have been proposed (30–34).

One of the most recurrent and central hypotheses in this regard is the presence of errors in the brain’s predictive processes (31, 35, 36). This concept aligns with current theories of active perception and predictive coding, which suggest that the brain does not operate as a passive system merely waiting for sensory input to generate a motor response. Instead, it relies on an internal model that continuously integrates information about the body, the environment, and the anticipated future state of both. This model is predictive in nature, allowing motor plans to be generated and executed based on pre-existing neural activity rather than creating new responses at every moment. This predictive mechanism enables an efficient system with reduced latency in response times, a critical feature for vestibular, postural, balance, and gaze control processes—essential for responding rapidly (and for example, avoiding falls) to a constantly changing environment.

A key aspect of this internal model is the representation of body position, not only in relation to gravity and inertial motion vectors (which would derive primarily from vestibular inputs) but also in relation to the surrounding environment. This function relies heavily on the extended brain spatial navigation network, which is responsible for constructing and maintaining this representation (23, 36–39).

Following this line of reasoning, we have previously proposed that a core feature of PPPD is a discrepancy between the brain’s internal model of body position in space and incoming sensory responses (9). This discrepancy would not due to errors in the sensory afferents themselves (as would be the case in bilateral or unilateral vestibulopathy), but rather to deficits in the creation, updating, and utilization of the internal model. This hypothesis reconciles with the fact that PPPD can develop in patients with fully functional peripheral vestibular organs (1).

We believe that the findings of this study support this hypothesis, as they depict PPPD patients exhibiting significant dysfunction in spatial navigation skills, particularly in allocentric navigation.

To begin with, We used a different cohort of patients to confirm that PPPD individuals have poorer spatial navigation compared to other vestibular disorders and matched control subjects (Figure 1). We emphasize the comparability of peripheral vestibular function between the PPPD and vestibular non-PPPD control groups, reinforcing the idea that sensory input quality does not account for the significantly worse spatial navigation performance in PPPD. While reduced vestibular sensory input does lead to some degree of spatial navigation impairment, as seen in CSE data from Figure 1, the magnitude of this impairment does not reach the level observed in PPPD patients.

### 4.2 Spatial navigation deficits in PPPD are not primarily driven by visuo-vestibular conflict

We initially hypothesized that conducting the vMWM challenge in a VR immersive setting would modify these results. When designing this experiment, we speculated that the VR modality might provide some level of vestibular cues, or that head and neck movements could contribute to environmental exploration while searching for the hidden target. We debated whether PPPD patients would compensate for potential visual processing difficulties by increasing exploratory behavior—leading to higher head rotation and, consequently, improved navigation performance—or, conversely, whether excessive sensory input would exacerbate sensory conflicts, particularly in complex visuo-vestibular spatial calculations, resulting in worse performance.

Thus, we were somewhat surprised to find that all assessed metrics and analyses yielded similar results across NI and VR modalities (Figures 2, 8, 9), suggesting that VR immersion did not significantly alter spatial navigation performance in PPPD patients. This lack of difference is particularly meaningful, as it indicates that the observed spatial navigation impairments in PPPD are unlikely to be driven by visuo-vestibular conflicts or sensory overload—both of which have been proposed as key mechanisms underlying other symptoms of PPPD.

Furthermore, we did not find significant differences between groups in terms of head kinematics (Figure 11). To further explore this, we considered whether the lack of significance could be attributed to sample size limitations rather than the absence of a real effect. We observed a non-significant trend in which both PPPD and vestibular non-PPPD patients exhibited reduced head movement compared to healthy volunteers. We cautiously interpret this as a potential common consequence of reduced peripheral vestibular sensory input, leading to decreased head movement behavior in both groups within the context of this task.

### 4.3 Entropy based analysis supports impairments in allocentric mapping

One of the most consistent findings across different analyses was that the impaired navigation performance of the PPPD group became significantly more pronounced under a predominantly allocentric navigational challenge. This was most evident in Figure 3, where entropy metrics revealed greater impairments in PPPD when allocentric navigation was required. Qualitative analyses of navigation path maps and navigation heatmaps (Figures 4, 5) further support this pattern. Moreover, when analyzing entropy in detail, we observed that not only did PPPD patients perform worse under allocentric demands, but the nature of this impairment appeared to be specifically related to deficits in acquiring, managing, or utilizing cognitive maps of the environment. Rather than general uncertainty or insecurity in the task itself, Figure 6 suggests that the primary deficit in PPPD is associated with errors in constructing and updating internal spatial representations of target locations.

From a methodological perspective, this is an opportune moment to acknowledge the differential utility of navigation metrics. Among the many available measures for assessing spatial navigation, CSE remained a robust metric for evaluating overall navigational ability, successfully distinguishing between clinical groups (Figure 1). However, entropy-based metrics—which quantify disorder and uncertainty in navigation—were more sensitive to detecting the specific nature of spatial impairments in PPPD. Interestingly, entropy was less effective in distinguishing the milder impairment observed in vestibular non-PPPD participants. This may align with our previous hypothesis that navigational deficits in PPPD are inherently more disorganized and chaotic, making entropy measures particularly well-suited for assessing the disorder present in their navigation strategies.

### 4.4 Increased gaze scanning as compensation to allocentric uncertainty

Regarding gaze behavior, we found a significantly higher level of gaze exploration across the visual scene in PPPD patients (Figures 8, 9). This behavior was not distinctly different between NI and VR modalities and did not reach statistical significance between Ego-Allocentric settings. Given that allocentric navigation strategies conceptually and experimentally rely on acquiring visual cue information from the environment, we consider these findings as additional support for the notion that PPPD is primarily characterized by an allocentric navigational impairment (37, 40, 41).

While the correlation analysis did not reach statistical significance in Figure 10, we chose to include this plot in our results as it visually represents our hypothesis that the relationship between navigation and visual information use in PPPD patients is functionally distinct from both vestibular non-PPPD and healthy controls. We suspect that during navigation, PPPD patients’ navigational systems respond to internal errors, uncertainties, and failures in locating the hidden target by triggering larger saccades and gaze redirection. This increased exploratory behavior may reflect an attempt to capture more visual information from the environment to compensate for an inadequate internal model, ultimately aiming to enhance data consistency and reliability. While this remains speculative, the trend observed is consistent with our broader hypothesis of predictive coding disruptions in PPPD, although we acknowledge that this finding may also reflect other mechanisms, including task-related anxiety or frustration, which might be more pronounced in PPPD.

On one hand, the increased exploratory gaze behavior in PPPD patients further reinforces the notion of an allocentric spatial navigation dysfunction in this population. On the other hand, and we propose this cautiously given the lack of strong statistical correlation, we suggest that enhanced exploratory gaze behavior—particularly in the context of a spatial navigation challenge such as the one in this study—could serve as a potential marker of internal model errors related to spatial predictive coding. This hypothesis warrants further investigation, as it may provide a novel approach to analyzing spatial predictive impairments in the context of functional dizziness and related disorders.

### 4.5 High functional heterogeneity within the PPPD group

Interestingly, while PPPD participants as a group showed clear impairments in spatial navigation and increased gaze exploration compared to both control groups, we observed a wide degree of individual variability within the PPPD group—particularly in gaze dispersion (Figure 9) and navigational performance (Figure 1). Some PPPD participants demonstrated performance levels close to those of the vestibular control group, suggesting the possibility of meaningful heterogeneity or even distinct cognitive subgroups within the PPPD population. This idea has been explored in more detail in a prior publication from our group using the same cohort (10), where we hypothesized that different cognitive profiles may underlie distinct patterns of dysfunction in PPPD. These findings align with the growing perspective that PPPD may not represent a single, well-bounded clinical entity, but rather a spectrum of functional dizziness presentations with varying degrees of spatial, cognitive, and sensorimotor involvement. Identifying and characterizing these potential subgroups will require exploratory methodologies and larger sample sizes in future research. In this regard, we believe that particular attention should be paid to the presence of comorbid vestibular migraine—especially its chronic variants—as this overlap may significantly contribute to the cognitive and functional heterogeneity observed in PPPD.

### 4.6 Virtual reality intolerance in PPPD/vestibular migraine comorbidity

Finally, we also observed a higher level of VR intolerance in both the PPPD and vestibular non-PPPD groups. We hypothesize that this may be strongly associated with the presence of vestibular migraine in both groups. This finding suggests that future research should include distinct groups of vestibular migraine patients without PPPD and PPPD patients without vestibular migraine, allowing for a more precise examination of visuo-vestibular conflict phenomena in visuospatial tasks such as this one. For example, this could help determine whether visual vertigo (which we associate with VR motion sickness in this study) is an independent symptom in both PPPD and vestibular migraine, or whether it is primarily a vestibular migraine phenomenon that becomes exacerbated when comorbid with PPPD—a well-documented clinical scenario (1, 42).

Nevertheless, given our current dataset and the similar proportion of vestibular migraine patients in both the PPPD and vestibular non-PPPD groups, we do not believe that vestibular migraine significantly affects our interpretation of spatial navigation impairments in PPPD.

### 4.7 Possible clinical implications of these findings

From a broader clinical perspective, the present findings raise the possibility that spatial navigation performance—and even gaze behavior during such tasks—could serve as potential behavioral biomarkers for PPPD. Although not intended as standalone diagnostic tools, these objective markers could eventually complement existing clinical criteria and contribute to multimodal diagnostic scoring systems for functional dizziness.

Moreover, the identification of allocentric spatial navigation impairments and increased exploratory gaze behavior in PPPD suggests that these cognitive features may represent therapeutic targets. This opens the door to integrating cognitive support strategies into balance and vestibular rehabilitation programs, particularly for those patients showing pronounced spatial or visual-cognitive dysfunction. In specific subgroups, cognitive rehabilitation focused on spatial processing—including the use of virtual navigation tasks or serious games—might offer a novel, tailored approach to treatment. Such strategies could not only address the spatial mapping deficits directly but also reduce the cognitive load and uncertainty that may sustain symptoms in PPPD.

Again, at this point these ideas are purely speculative, and future research could aim to validate these findings in larger samples and determine whether navigation-based training could improve both objective performance and subjective symptom burden in functional dizziness.

## 5 Conclusion

The analysis of gaze behavior, head kinematics, and spatial navigation performance—including metrics such as entropy—in both NI and VR experimental settings supports the notion that a core feature of PPPD is an impairment in allocentric spatial navigation processes. Moreover, entropy-based metrics proved particularly sensitive to the disorganized nature of PPPD-related navigational behavior.

Importantly, the fact that navigation performance remained consistent across NI and VR modalities suggests that visuo-vestibular conflict and sensory overload are not the primary drivers of these deficits, challenging some commonly held assumptions in PPPD pathophysiology. Instead, the observed increased gaze exploration in PPPD patients may represent a compensatory response to internal model uncertainty, and thus serve as a potential behavioral marker of impaired spatial predictive coding.

We hope that these findings not only contribute to the development of diagnostic and therapeutic tools for PPPD but also offer a broader perspective on functional neurological disorders, conceptualizing them as discrepancies between internal models of the body and the surrounding world versus real sensory inputs—where the underlying dysfunction may lie in the former rather than the latter.

## Data Availability

The datasets presented in this study can be found in online repositories. The name of the repository and accession number can be found at: https://www.labonce.cl/s-projects-basic.

## References

[1] Staab JP Eckhardt-Henn A Horii A Jacob R Strupp M Brandt T. Diagnostic criteria for persistent postural-perceptual dizziness (PPPD): consensus document of the committee for the classification of vestibular disorders of the Bárány society. J Vestib Res. (2017) 27:191–208. doi: 10.3233/VES-170622, PMID: 29036855 PMC9249299

[2] Madrigal J Herrón-Arango AF Bedoya MJ Chen JC Castillo-Bustamante M. Persistent challenges: a comprehensive review of persistent postural-perceptual dizziness, controversies, and clinical complexities. Cureus. (2024) 16:e60911. doi: 10.7759/CUREUS.60911, PMID: 38910644 PMC11193666

[3] Staab JP. Persistent Postural-Perceptual Dizziness. Neurol Clin. (2023) 41:647–64. doi: 10.1016/j.ncl.2023.04.003 37775196

[4] Dieterich M Staab JP. Functional dizziness: From phobic postural vertigo and chronic subjective dizziness to persistent postural-perceptual dizziness. Curr Opin Neurol. (2017) 30:107–13. doi: 10.1097/WCO.0000000000000417, PMID: 28002123

[5] Staab JP. Persistent Postural-Perceptual Dizziness. Semin Neurol. (2020) 40:130–7. doi: 10.1055/s-0039-3402736 31935771

[6] Dieterich M Staab JP Thomas B. Functional (psychogenic) dizziness. Handb Clin Neurol. (2016) 139:447–68. doi: 10.1016/B978-0-12-801772-2.00037-0, PMID: 27719862

[7] Cousins S Kaski D Cutfield N Arshad Q Ahmad H Gresty MA. Predictors of clinical recovery from vestibular neuritis: a prospective study. Ann Clin Transl Neurol. (2017) 4:340–6. doi: 10.1002/acn3.386, PMID: 28491901 PMC5420806

[8] Yagi C Kimura A Horii A. Persistent postural-perceptual dizziness: A functional neuro-otologic disorder. Auris Nasus Larynx. (2024) 51:588–98. doi: 10.1016/J.ANL.2023.12.008, PMID: 38552422

[9] Breinbauer HA Contreras MD Lira JP Guevara C Castillo L Ruëdlinger K. Spatial navigation is distinctively impaired in persistent postural perceptual dizziness. Front Neurol. (2020) 10:1361. doi: 10.3389/fneur.2019.01361, PMID: 31998220 PMC6970195

[10] Breinbauer HA Arévalo-Romero C Villarroel K Lavin C Faúndez F Garrido R. Functional dizziness as a spatial cognitive dysfunction. Brain Sci. (2023) 14:16. doi: 10.3390/BRAINSCI14010016 38248231 PMC10813051

[11] Arshad Q Saman Y Sharif M Kaski D Staab JP. Magnitude estimates orchestrate hierarchal construction of context-dependent representational maps for vestibular space and time: theoretical implications for functional dizziness. Front Integr Neurosci. (2022) 15:940. doi: 10.3389/fnint.2021.806940, PMID: 35185485 PMC8855482

[12] Arshad I de Mello P Ender M McEwen JD Ferré ER. Reducing Cybersickness in 360-degree virtual reality. Multisens Res. (2021) 16:1–17. doi: 10.1163/22134808-BJA10066 34936982

[13] Tarnutzer AA Kaski D. What’s in a name? Chronic vestibular migraine or persistent postural perceptual dizziness? Brain Sci. (2023) 13:1692. doi: 10.3390/BRAINSCI13121692 38137140 PMC10741489

[14] Lempert T Olesen J Furman J Waterston J Seemungal B Carey J. Vestibular migraine: diagnostic criteria. J Vestib Res. (2012) 22:167–72. doi: 10.3233/ves-2012-0453, PMID: 23142830

[15] Lopez-Escamez JA Carey J Chung W-H Goebel JA Magnusson M Mandala M. Diagnostic criteria for Meniere’s disease. J Vestib Res. (2015) 25:1–7. doi: 10.3233/VES-150549, PMID: 25882471

[16] von Brevern M Bertholon P Brandt T Fife T Imai T Nuti D. Benign paroxysmal positional vertigo: Diagnostic criteria. J Vestib Res. (2015) 25:105–17. doi: 10.3233/ves-150553, PMID: 26756126

[17] Strupp M Kim J-S Murofushi T Straumann D Jen JC Rosengren SM. Bilateral vestibulopathy: diagnostic criteria consensus document of the classification Committee of the Barany Society. J Vestib Res. (2017) 27:177–89. doi: 10.3233/VES-170619, PMID: 29081426 PMC9249284

[18] Schoenfeld R Schiffelholz T Beyer C Leplow B Foreman N. Variants of the Morris water maze task to comparatively assess human and rodent place navigation. Neurobiol Learn Mem. (2017) 139:117–27. doi: 10.1016/j.nlm.2016.12.022, PMID: 28057502

[19] Wood H. A virtual Morris maze to assess cognitive impairment in Alzheimer disease. Nat Rev Neurol. (2016) 12:126–6. doi: 10.1038/nrneurol.2016.16, PMID: 26846453

[20] Wei EX Oh ES Harun A Ehrenburg M Agrawal Y. Vestibular loss predicts poorer spatial cognition in patients with Alzheimer’s disease. J Alzheimers Dis. (2018) 61:995–1003. doi: 10.3233/JAD-170751, PMID: 29254098

[21] Dobbels B Mertens G Gilles A Moyaert J van de Berg R Fransen E. The virtual Morris water task in 64 patients with bilateral Vestibulopathy and the impact of hearing status. Front Neurol. (2020) 11:1–12. doi: 10.3389/fneur.2020.00710, PMID: 32849193 PMC7431773

[22] Dobbels B Peetermans O Boon B Mertens G Van de Heyning P Van Rompaey V. Impact of bilateral Vestibulopathy on spatial and nonspatial cognition: a systematic review. Ear Hear. (2019) 40:757–65. doi: 10.1097/AUD.0000000000000679, PMID: 31242136

[23] Kremmyda O Hüfner K Flanagin VL Hamilton DA Linn J Strupp M. Beyond dizziness: virtual navigation, spatial anxiety and hippocampal volume in bilateral Vestibulopathy. Front Hum Neurosci. (2016) 10:139. doi: 10.3389/fnhum.2016.00139, PMID: 27065838 PMC4814552

[24] Pereira IT Burwell RD. Using the spatial learning index to evaluate performance on the water maze. Behav Neurosci. (2015) 129:533–9. doi: 10.1037/bne0000078, PMID: 26214218 PMC5077721

[25] Possin KL Sanchez PE Anderson-Bergman C Fernandez R Kerchner GA Johnson ET. Cross-species translation of the Morris maze for Alzheimer’s disease. J Clin Invest. (2016) 126:779–83. doi: 10.1172/JCI78464, PMID: 26784542 PMC4731157

[26] Maei H. Development and validation of a sensitive entropy-based measure for the water maze. Front Integr Neurosci. (2009) 3:33. doi: 10.3389/neuro.07.033.2009, PMID: 20057926 PMC2802531

[27] Maei HR. What is the most sensitive measure of water maze probe test performance? Front Integr Neurosci. (2009) 3:4. doi: 10.3389/neuro.07.004.2009, PMID: 19404412 PMC2659169

[28] Im JJ Na S Jeong H Chung YA. A review of neuroimaging studies in persistent postural-perceptual dizziness (PPPD). Nucl Med Mol Imaging. (2021) 55:53–60. doi: 10.1007/s13139-020-00675-2, PMID: 33968271 PMC8053630

[29] Storm R Krause J Blüm SK Wrobel V Frings A Helmchen C. Visual and vestibular motion perception in persistent postural-perceptual dizziness (PPPD). J Neurol. (2024) 271:3227–38. doi: 10.1007/s00415-024-12255-x, PMID: 38441610 PMC11136745

[30] Regnath F Biersack K Schröder L Stainer MC von Werder D Pürner D. Experimental evidence for a robust, transdiagnostic marker in functional disorders: erroneous sensorimotor processing in functional dizziness and functional movement disorder. J Psychosom Res. (2024) 183:111694. doi: 10.1016/J.JPSYCHORES.2024.111694, PMID: 38734533

[31] Aybek S Perez DL. Diagnosis and management of functional neurological disorder. BMJ. (2022) 376:64. doi: 10.1136/bmj.o64, PMID: 35074803

[32] Cretton A Brown RJ Lafrance WC Aybek S. What does neuroscience tell us about the conversion model of functional neurological disorders? J Neuropsychiatry Clin Neurosci. (2020) 32:24–32. doi: 10.1176/APPI.NEUROPSYCH.19040089, PMID: 31619119

[33] Edwards MJ. Functional neurological disorder: an ethical turning point for neuroscience. Brain. (2019) 142:1855–7. doi: 10.1093/BRAIN/AWZ194, PMID: 31505545

[34] Jungilligens J Paredes-Echeverri S Popkirov S Barrett LF Perez DL. A new science of emotion: implications for functional neurological disorder. Brain. (2022) 145:2648–63. doi: 10.1093/brain/awac204, PMID: 35653495 PMC9905015

[35] Clark A. Whatever next? Predictive brains, situated agents, and the future of cognitive science. Behav Brain Sci. (2013) 36:181–204. doi: 10.1017/S0140525X12000477, PMID: 23663408

[36] Do TTN Lin CT Gramann K. Human brain dynamics in active spatial navigation. Sci Rep. (2021) 11:13036. doi: 10.1038/s41598-021-92246-4, PMID: 34158525 PMC8219750

[37] Epstein RA Patai EZ Julian JB Spiers HJ. The cognitive map in humans: spatial navigation and beyond. Nat Neurosci. (2017) 20:1504–13. doi: 10.1038/nn.4656, PMID: 29073650 PMC6028313

[38] Patai EZ Spiers HJ. The versatile Wayfinder: prefrontal contributions to spatial navigation. Trends Cogn Sci. (2021) 25:520–33. doi: 10.1016/J.TICS.2021.02.010, PMID: 33752958

[39] Biju K Wei EX Rebello E Matthews J He Q McNamara TP. Performance in real world-and virtual reality-based spatial navigation tasks in patients with vestibular dysfunction. Otol Neurotol. (2021) 42:E1524–31. doi: 10.1097/MAO.0000000000003289, PMID: 34766948

[40] Ekstrom AD Arnold AEGF Iaria G. A critical review of the allocentric spatial representation and its neural underpinnings: toward a network-based perspective. Front Hum Neurosci. (2014) 8:803. doi: 10.3389/fnhum.2014.00803, PMID: 25346679 PMC4193251

[41] Abedi Khoozani P Bharmauria V Schutz A Wildes RP Crawford JD. Integration of allocentric and egocentric visual information in a convolutional/multilayer perceptron network model of goal-directed gaze shifts. Cereb Cortex. (2022) 3:26. doi: 10.1093/texcom/tgac026, PMID: 35909704 PMC9334293

[42] Chang TP Hong YC Schubert MC. Visual vertigo and motion sickness is different between persistent postural-perceptual dizziness and vestibular migraine. Am J Otolaryngol. (2024) 45:104321. doi: 10.1016/J.AMJOTO.2024.104321, PMID: 38696894

