# Supplementary material for: Spatial navigation entropy suggests allocentric dysfunction in PPPD
Source: Front Neurol. 2025 May 16;16:1599307. doi: 10.3389/fneur.2025.1599307 (PMC12122347; doi:10.3389/fneur.2025.1599307)
Supplement: Supplementary file 1 [file Supplementary_file_1.docx]

Supplementary Material

# Detailed Description of the vMWM Protocol

A full account of the six-block Virtual Morris Water Maze (vMWM) protocol used in this study is provided below. Each block was designed with specific methodological purposes, including participant familiarization, visual cue manipulation, and control of potential biases such as fatigue and motor difficulties. While the main analysis in the manuscript focuses on Blocks C and E, the full structure of the protocol is essential for understanding the design's internal consistency and reliability. Prior data from this cohort validating these control conditions have been published previously (Ref. 10).

Our vMWM testing protocol was essentially the same for both the NI and VR modalities. To minimize learning bias, we changed the target positions across settings. The overall structure of the protocol consisted of six blocks, described as follows:

***Block A***: consisted of two training trials: (i) free navigation was allowed for familiarization with joystick movements; (ii) participants were instructed to navigate towards the visual cues on the room’s walls as a practice exercise.

***Block B*:** encompassed four consecutive trials. A visible red square platform, measuring 0.17 × 0.17 virtual units was consistently located in the pool’s north-eastern quadrant. Participants initiated each trial at the southern end of the pool, facing north. Successful arrival at the platform was indicated by a rewarding sound, signaling trial completion. This block was designed to familiarize participants with the virtual environment and the test protocol. The maximal trial duration was set to 1 min for this and all subsequent blocks, irrespective of whether the target was reached or not. Blocks A and B serve as initial tests to identify any motor or group-specific issues related to manipulating the joystick and navigating within the virtual environment. We have chosen to include data from these blocks to emphasize that differences observed in subsequent blocks are indeed attributable to variations in navigational skills and not influenced by other potential con-founding factors.

***Block C*:** included seven consecutive trials. Starting from the same southern location, participants were tasked with locating a hidden platform situated in the south-western quadrant. This platform became visible only upon direct contact and emitted the same rewarding sound. Participants were instructed to remember and optimize their route to the platform across the trials. The visual cues provided were consistent: old-style airplanes (north), a sea turtle (west), “La Gioconda” by Leonardo da Vinci (south), and flowers (east). This phase represented the initial setting that necessitated the application of either egocentric or allocentric spatial navigation strategies. Participants were required to memorize the location of the hidden platform and utilize these strategies to efficiently locate it in subsequent trials.

***Block D*:** was a mirrored version of Block C, comprising another set of seven trials. Here, the hidden platform was relocated to the north-western quadrant. The visual cues were changed to simple colored geometric symbols: a black cross (north), a red square (west), a pink heart (south), and a blue triangle (east). Block D closely resembles the preceding block, with the distinction of employing visual cues of markedly lower complexity, devoid of any emotional connotation. These cues consisted of simple geometric symbols as opposed to the more evocative animals or artworks used previously. The aim of this design choice was to evaluate the potential emotional influence exerted by the visual cues on the navigation process.

***Block E:*** involved a sequence of seven trials similar to Blocks C and D. The hidden platform was positioned on the pool’s western side. New visual cues featured a flying condor bird (north), a sunflower field (west), Van Gogh’s “La Méridienne” (south), and an old train (east). This block introduced an increased spatial navigation challenge by incorporating random starting positions and initial facing orientations. Typically, such a “random start” setting is believed to maximize the reliance on allocentric navigational mechanisms [53].

***Block F*:** consisted of four trials, similar in complexity to Block B, with a distinctly visible platform positioned in the south-east quadrant. It employed random starting points, as in Block E, to serve as a control for the assessment of joystick manipulation and movement within the virtual environment.

Blocks A, B, and F were designed to assess the reliability and potential biases of our protocols. These blocks are intended to ensure that all subjects perform equally well across them; any significant differences could indicate a more fundamental motor issue, an experimental setting problem, or variability unrelated to spatial navigation performance, thereby compromising the ability to assess higher cognitive functions such as spatial navigation across groups. Furthermore, no significant differences are expected between Blocks B and F, as this would suggest fatigue effects during the assessment, which could compromise the interpretation of subsequent findings.

Data regarding these blocks for the same cohort analyzed in this study were previously explored in a separate publication (10), which confirmed no major differences across groups or between Blocks B and F. Additionally, as reported in the same study (Reference 10 on main text), no significant differences were observed between Blocks C and D, where the only variation was the type of visual cue used.

Consequently, in this study, and to avoid overwhelming the reader with redundant figures and data, we focus our analysis on Blocks C and E. These blocks share similar visual cues, but differ in their starting conditions. In Block C, participants always start from a fixed location, allowing both egocentric and allocentric strategies to be employed. In contrast, Block E introduces variability by having participants start from a random position and orientation within the pool, reducing their ability to rely on egocentric cues and preferentially testing allocentric spatial navigation skills. For clarity, we refer to Block C as the ***“Ego-Allocentric Setting”*** and Block E as the ***“Mainly Allocentric Setting.”***

# Vestibular testing and imaging (detailed)

## Vestibular Function

To evaluate vestibular function and ascertain vestibular dysfunction in the test and control groups, video head impulse testing (vHIT) and vestibular evoked myogenic potentials (VEMPs) were administered to all participants.

### Video Head Impulse Testing (vHIT)

The vestibulo-ocular reflex (VOR) function of all six semicircular canals was evaluated using the ICS impulse video head impulse testing (vHIT) device by GN Otometrics, Denmark. A total of 20 head impulses were administered per canal. Impulses were deemed valid if they were free of recognizable artifacts and achieved a peak head velocity exceeding 200°/s for the lateral canals and 150°/s for the vertical canals.

### Vestibular Evoked Myogenic Potentials (VEMPs)

For recording vestibular evoked myogenic potentials (VEMPs), both ocular (oVEMP) and cervical (cVEMP) responses were obtained using the Eclipse EP25 platform. Standard protocols were followed: oVEMP tests required subjects to sit and gaze upwards at a 30° angle, while cVEMP tests were conducted with the subject supine and the head raised for sternocleidomastoid muscle contraction, monitored for consistent electromyographic signals. A 500 Hz tone burst at 100 dB nHL was used for air-conduction stimulation, with over 100 stimuli per ear averaged to ensure arti-fact-free, reproducible responses.

## Magnetic Resonance Imaging

Images were acquired at the “Servicio de Resonancia Magnética y Tomografía Computada de la Clínica Alemana de Santiago” using a 3T Siemens SKYRA MRI, Berlin, Germany, system. A neuroradiologist, who was blinded to the clinical evaluations of both volunteers and patients, reviewed and interpreted the images. For this study, the images were specifically examined to rule out additional diseases or any form of structural damage in brain regions crucial for spatial navigation, including the hippocampus.

# Supplementary Figures and Tables

**Table S1.** Vestibular function across PPPD, vestibular, and control groups**.**

|  |  |  | **Group** | | | **Statistical Testing between Groups** | | |
| --- | --- | --- | --- | --- | --- | --- | --- | --- |
|  | **Ear** | **Canal** | **PPPD** | **Vestibular**  **(Non-PPPD)** | **Healthy Control** | **ANOVA**  **(F; *p*-Value)** | **Specific Groups Presenting Significant Difference after Post Hoc Tukey (*p*-Value <0.05)** | **Post Hoc Tukey (*p*-Value)** |
| Video Head Impulse Test (vHIT) | Right | Anterior | **0.88** (0.1)  *Gain <0.7:* ***6.3%***  *Sacc: 0%* | **0.9** (0.10)  *Gain <0.7:* ***8.5%***  *Sacc:* ***0%*** | **0.91** (0.07)  *Gain <0.7:* ***0%***  *Sacc:* ***0%*** | F = 0.16;  *p* = 0.85 |  |  |
|  |  | Lateral | **0.92** (0.08)  *Gain <0.7****: 5.2%***  *Sacc:* ***21%*** | **0.94** (0.07)  *Gain <0.7:* ***0%***  *Sacc:* ***20%*** | **0.96** (0.02)  *Gain <0.7:* ***0%***  *Sacc:* ***0%*** | F = 0.19;  *p* = 0.82 |  |  |
|  |  | Posterior | **0.85** (0.07)  *Gain <0.7:* ***20%***  *Sacc:* ***11%*** | **0.88** (0.08)  *Gain <0.7:* ***26.2%***  *Sacc:* ***25%*** | **0.92** (0.03)  *Gain <0.7:* ***0%***  *Sacc:* ***0%*** | F = 4.38;  *p* = 0.038 | PPPD < Healthy Control  Vestibular < Healthy Control | *p* = 0.042  *p* = 0.048 |
| Gain:  **mean** (standard deviation)  *(Gain <0.7: %) Percentage of patients exhibiting gain lower than 0.7*  *(Sacc: %) Percentage of patients exhibiting corrective saccades* | Left | Anterior | **0.87** (0.08)  *Gain <0.7:* ***21%***  *Sacc:* ***11%*** | **0.86** (0.07)  *Gain <0.7:* ***10%***  *Sacc:* ***0%*** | **0.9** (0.08)  *Gain <0.7:* ***8.3%***  *Sacc:* ***0%*** | F = 0.86; *p* = 0.42 |  |  |
|  |  | Lateral | **0.86** (0.08)  *Gain <0.7:* ***23.3%***  *Sacc:* ***37%*** | **0.85** (0.11)  *Gain <0.7:* ***20%***  *Sacc:* ***25%*** | **0.91**(0.03)  *Gain <0.7:* ***0%***  *Sacc:* ***0%*** | F = 3,92;  *p* = 0.031 | PPPD < Healthy Control  Vestibular < Healthy Control | *p* = 0.041  *p* = 0.044 |
|  |  | Posterior | **0.92** (0.09)  *Gain <0.7:* ***10.5%***  *Sacc:* ***11%*** | **0.90** (0.13)  *Gain <0.7:* ***15%***  *Sacc:* ***10%*** | **0.93** (0.05)  *Gain <0.7:* ***0%***  *Sacc:* ***0%*** | F = 0.03; *p* = 0.96 |  |  |
|  | Ear with  higher gain | Anterior | **0.9** (0.09)  *Gain <0.7:* ***8%***  *Sacc:* ***0%*** | **0.92** (0.07)  *Gain <0.7:* ***5%***  *Sacc:* ***0%*** | **0.94** (0.03)  *Gain <0.7:* ***0%***  *Sacc:* ***0%*** | F = 0.36; *p* = 0.69 |  |  |
|  |  | Lateral | **0.93** (0.08)  *Gain <0.7:* ***5%***  *Sacc:* ***21%*** | **0.94** (0.07)  *Gain <0.7:* ***8.5%***  *Sacc:* ***25%*** | **0.93** (0.02)  *Gain <0.7:* ***0%***  *Sacc:* ***0%*** | F = 0.16; *p* = 0.84 |  |  |
|  |  | Posterior | **0.94** (0.08)  *Gain <0.7:* ***5.2%***  *Sacc:* ***10%*** | **0.97** (0.09)  *Gain <0.7:* ***0%***  *Sacc:* ***0.5%*** | **0.94** (0.04)  *Gain <0.7:* ***0%***  *Sacc:* ***0%*** | F = 0.62; *p* = 0.54 |  |  |

Although not the main focus of this manuscript, a detailed analysis of vestibular function was essential to confirm the comparability of each group. Table A1 presents a data of vestibular function across all groups. The data are initially displayed for each ear. To enhance comparability, results are also shown for the ear exhibiting the best performance and the one with the poorest performance, thus mitigating bias from patients with unilateral vestibular disease. For the vHIT data, the mean vestibulo-ocular reflex (VOR) gain and its standard deviation are provided for each ear and canal. Additionally, the proportion of patients displaying a gain lower than 0.7 and the proportion of patients presenting pathological corrective saccades within each group are depicted. The PPPD and vestibular groups exhibit comparable levels of function/dysfunction, both showing reduced function relative to healthy controls, particularly when examining the lateral and posterior canals of the ear with the poorest performance (specific intergroup differences were tested using ANOVA with a Tukey post hoc analysis, yielding a *p*-value less than 0.05).

A parallel analysis is conducted for VEMP amplitude responses, with data presented for each ear and then for the ear with the best/worst performance. For oVEMP, the ear with the poorest performance demonstrated differences between both the PPPD and vestibular groups compared to healthy controls, but no distinction was noted between the PPPD and vestibular groups themselves.

Regarding cVEMPs, statistical significance was observed only in the poorer-performing ear between the vestibular group and healthy controls. In summary, as anticipated, both the PPPD and vestibular groups, possessing vestibular pathologies, exhibited inferior vestibular function compared to healthy volunteers. However, no significant difference between the PPPD and vestibular groups was identified in terms of vestibular function.
